# Zoonotic potential and prevalence of *Salmonella* serovars isolated from pets

**DOI:** 10.1080/20008686.2021.1975530

**Published:** 2021-09-08

**Authors:** Mateusz Dróżdż, Michał Małaszczuk, Emil Paluch, Aleksandra Pawlak

**Affiliations:** a Freie Universität Berlin, Institute of Chemistry and Biochemistry, Laboratory of Rna Biochemistry, Berlin, Germany; b Department of Microbiology, University of Wroclaw, Wrocław, Poland; c Department of Microbiology, Faculty of Medicine, Wroclaw Medical University, Wrocław, Poland

**Keywords:** Pet animals, zoonotic transmission, *salmonella* serovars distribution, pet regulations

## Abstract

Salmonellosis is a global health problem, affecting approximately 1.3 billion people annually. Most of these cases are related to food contamination. However, although the majority of *Salmonella* serovars are pathogenic to humans, animals can be asymptomatic carriers of these bacteria. Nowadays, a wide range of animals is present in human households as pets, including reptiles, amphibians, dogs, cats, ornamental birds, and rodents. Pets contaminate the environment of their owners by shedding the bacteria intermittently in their feaces. In consequence, theyare thought to cause salmonellosis through pet-to-human transmission. Each *Salmonella* serovar has a different zoonotic potential, which is strongly regulated by stress factors such as transportation, crowding, food deprivation, or temperature. In this review, we summarize the latest reports concerning *Salmonella*-prevalence and distribution in pets as well as the risk factors and means of prevention of human salmonellosis caused by contact with their pets. Our literature analysis (based on PubMed and Google Scholar databases) is limited to the distribution of *Salmonella* serovars found in commonly owned pet species. We collected the recent results of studies concerning testing for *Salmonella* spp. in biological samples, indicating their prevalence in pets, with regard to clinical cases of human salmonellosis.

## Background

Keeping pets provides health, emotional and social benefits to their owners. According to the American Pet Products Association’s 2019–2020 National Pet Owners Survey, approximately 67% of households and 85 million families own at least one pet [1,2]. It was estimated that 63.4 million and 42.7 million households own dogs and cats, respectively, with 5.7 million and 4.5 million pet owners keeping ornamental birds and reptiles [3]. In contrast, the European Pet Food Industry reported that cats are the most popular pets in Europe (about 110,000,000 in 2020), followed by dogs (about 90,000,000 in 2020) and ornamental birds (52,000,000 in 2020). Furthermore, in Europe, the pet-reptile population is approximately 9 000 000, with the UK ranked as the top country [4].

In spite of the beneficial effects of pets on human’s health, these animals may be asymptomatic carriers of different bacterial (e.g., *Pasteurella, Salmonella, Brucella, Yersinia, Campylobacter, Capnocytophaga, Coxiella, Leptospira, Chlamydia)* [5,6], fungal (e.g., *Candida* sp. *Aspergillus* sp.) [7], parasitic (e.g., arthropods, helminths, protozoa) [8], and viral (e.g., rabies, norovirus, lymphocytic choriomeningitis virus) pathogens. Salmonellosis is one of the most serious zoonotic diseases in the world. Its etiologic agents – *Salmonella* spp. are in most cases pathogenic for people affecting primarily young children (<5 years), older adults (age >64 years), immunocompromised people, and pregnant women [9]. For some animals, *Salmonella* spp. is thought to be an opportunistic pathogen or even a part of natural gut microbiota. For instance, up to 80–90% of reptiles are asymptomatic *Salmonella* spp carriers [10–13]. It is well documented that reptiles may lead to cases of human salmonellosis, and these infections gained a separate disease entity and abbreviation – reptile associated salmonellosis (RAS) and reptile-exotic-pet associated salmonellosis (REPAS) to indicate the role of domestic reptiles living at households in spreading a *Salmonella* spp [14].

## Methods

In order to address the globally increasing pet-to-human transmission of *Salmonella* spp., it is crucial to establish the background of *Salmonella*-prevalence and their distribution in pets. This review aimed to describe the epidemiology of pet-associated salmonellosis and determine the retail sources of pets linked to human illness. We identified primary literature, reviews, and consensus guidelines through the National Library of Medicine’s PubMed using the following search terms: *’Salmonella’* or ’salmonellosis’ AND ’pets’ or ’companion animals’ or ’zoonosis’ or ’zoonotic infection’ or ’amphibians’ or ’reptiles’ or ’dogs’ or ’cats’ or ’ornamental birds’ or ’rodents’ or ’guinea pigs’ or ’international trade’ or ’diet’. In order to provide pivotal and novel insights into the topic of zoonotic salmonellosis linked to the contact with companion animals and including the increasing in recent years tendency to keep exotic animals in households, we mainly focused on articles published in the second half of the last decade (2015–2020). However, the existing literature included in this review has been actualized by performing a literature search to add new relevant publications published in 2021. Original articles in English and different national languages (Polish, French, Spanish, if available) were included. Articles were screened by reading titles and abstracts and were initially excluded if they did not refer to zoonotic salmonellosis or were related to human salmonellosis caused by food or water contamination or due to human-to-human transmission. Articles were then read in full, especially those aiming to detect *Salmonella* spp. samples in the feaces of different companion animals and clinical cases of *Salmonella* infection linked to contact with them (for instance, RAS salmonellosis). If a selected article was a review, we searched for relevant citations to find primary literature on the subject. Occasionally, reviews were directly used as sources, mainly to convey background information that is not in the core focus of this review or to additionally confirm the usage of data from a specific citation. We identified more than 500 articles of interest, of which we included 147 articles in this review based on their content. Furthermore, if the additional information was not available in scientific publication, we referred to the internet. We sorted out all the crucial information that we were willing to provide in this review in the following order, recapitulating the number of available literature in this scientific area: amphibians and reptiles, dogs, cats, pet birds and pet rodents (18 articles for ‘*Salmonella*’ and ‘amphibians’, 156 articles for ‘*Salmonella*’ and ‘reptiles’, 114 articles for ‘*Salmonella*’ and ‘dogs’, 46 articles for ‘*Salmonella*’ and ‘cats’, 15 articles for ‘*Salmonella*’ and ‘pet birds’, and 11 articles for ‘*Salmonella*’ and ‘pet rodents’ in the last 6 years searched in the PubMed database).

## Classification of *Salmonella* spp

*Salmonella* spp. are a global problem of public health, as they cause almost 1.3 billion cases of illness each year, leading to more than 3 million deaths [15]. In the USA (US) alone, approximately 1.2 million human infections, 23,000 hospitalizations and 450 deaths occur each year. In contrast, in European Union (EU) countries, salmonellosis is the second most commonly reported gastrointestinal infection, followed by *Campylobacter* sp. In 2018, approximately 92,000 confirmed cases [16] of human salmonellosis were documented. Including total notification of human salmonellosis for the last 6 years, the stabilized tendency after a long period of a declining trend is observed [16].

*Salmonella spp*. are Gram-negative, rod-shaped bacteria belonging to the family *Enterobacteriaceae*, order *Enterobacterales*. The genus *Salmonella* is divided into two broad species named *Salmonella enterica* and *Salmonella bongori. S. enterica* consists of six subspecies: *enterica* (I), *salamae* (II), *arizonae* (IIIa), *diarizonae* (IIIb), *houtenae* (IV) and *indica* (VI) [17]. According to the last published supplement (no. 48–2014) of White-Kauffmann-Le Minor scheme, 2659 *Salmonella* serovars were identified [18]. Including the distribution of *S. enterica*, a great number of serovars (1586) are found in subsp. *enterica* that are responsible for more than 99% of human salmonellosis. Other *Salmonella enterica* serovars are unevenly distributed among the following subspecies: *salamae –* 522 serovars; *diarizonae* – 338 serovars; *arizonae –* 102 serovars; *houtenae* – 76 serovars and *indica* – 13 serovars [19,20].

## Typhoidal salmonellosis

Based on the ability to develop specific pathologies in humans, all known *Salmonella* serovars are classified into typhoidal and non-typhoidal salmonellosis. Typhoidal *Salmonella* serovars including Typhi, Sendai and Paratyphi are highly adapted to humans; animals are not their carriers. These pathogens are the causative agents of enteric fever (also known as typhoid or paratyphoid fever if caused by serovar, Typhi or Paratyphi, respectively). This disease is characterized by low morbidity and high mortality displaying several symptoms, such as high fever, diarrhea, vomiting and headache [21]. Worldwide, enteric fever is the most prevalent in impoverished areas that are overcrowded with poor access to sanitation. To date, the highest incidences of typhoidal *Salmonella* infection in the world occurred in south-central Asia, southeast Asia, and southern Africa [22].

## Non-typhoidal salmonellosis

Non-typhoidal salmonellosis (NTS) is a zoonotic disease caused by multiple *Salmonella* serovars other than Typhi, Sendai, and Paratyphi. Due to differential disease symptoms, NTS can be divided into non-invasive and invasive (iNTS). The vast majority of the non-invasive NTS proceed as gastrointestinal self-limiting infections and do not require antibiotic treatment [23]. Invasive salmonellosis is a more severe disease with sepsis, septic aortic aneurysm, and septic arthritis, meningitidis, and are thought to result in the patient’s death. Most of the iNTS are caused by the same serovars as non-invasive infections and affect people at higher risk group as children and elderly, people with health defects (AIDS or liver cirrhosis patients) and pregnant women; antimicrobial treatment is always needed. Contact with pets can result in both non-iNTS and iNTS [24]. In general, NTS salmonellosis is considered as a foodborne disease (about 80% of all cases were caused by food contamination, reaching 94% in the US in 2012 [25,26]). According to reports from EFSA (European Food Safety Authority) and ECDC (European Centre for Disease Prevention and Control), there are more than 90,000 of NTS cases in Europe annually, with the highest number in Germany, Czech Republic, UK, and Poland [16,27]. It is worth noting that non-typhoidal salmonellosis cases are still underestimated as some mild infections are unreported. Also, the epidemiological investigation is not always properly conducted or not conducted at all.

## Routes of *Salmonella* spp. transmission

It was estimated that about 9% of human salmonellosis is caused by direct contact with animals. Considering only pets, these cases are significantly lower, accounting for approximately 1% of morbidity of human salmonellosis per year [28]. As *Salmonella* spp.are assummed to belong to the natural microbiota of animal’s intestine or gallbladder [29], these animals may also potentially lead to indirect or direct transmission of the pathogen to human. Pets may contaminate the environment and other food-producing animals by shedding the bacteria intermittently in their faeces [30]. Thus, *Salmonella* spp. is thought to spread by fecal-oral route during consumption of contaminated food or water. Stress factors such as transportation, mixing or crowding, food deprivation, parturition, exposure to cold, concurrent viral or parasitic disease, sudden change of feed or overfeeding, can lead to an increase in shedding load of *Salmonella* spp. to the environment [31]. For instance, De Lucia *et al*. [32] showed evidences of increased *Salmonella* spp. shedding by insufficient separation of wild birds from one outdoor pig farm. *Salmonella* spp. was isolated from pig faeces, environmental samples and wild bird droppings. The wild bird population increased considerably once the pigs had left the farm and the proportion of *Salmonella*-positive wild bird droppings increased over time with 7.4%, 15.8% and 44.3% at the first, second and third visit, respectively. The levels of *Salmonella* spp. identified in some of the wild bird droppings were high (10^5^–10^6^ CFU (colony-forming unit)/gram (g)) indicating that this pathogen was actively replicating in the gastrointestinal tract of wild birds, leading to soil and outdoor pig farm contamination [32]. Furthermore, fomites such as houseflies *(Musca domestica)* can also spread *Salmonella* spp. For instance, in the US, Xu *et al*. [33] determined *Salmonella*-prevalence in flies captured from 33 cattle farms, including 5 beef and 28 dairy farms, and characterized antibiotic resistance profiles of the isolated *Salmonella* spp. 26 out of the 33 cattle farms (79%) and 185 out of the 1650 flies (11%) tested *Salmonella*-positive. These incidences varied from farm to farm, ranging from 0% to 78%, suggesting that flies are thought to be effective vehicles of transmitting antibiotic resistant *Salmonella* spp, posing risks to both human and animal health [33]. Another route, in which *Salmonella* spp. may spread is through vertical transmission, a phenomenon that occurs commonly in birds and reptiles. Avian eggs can be contaminated on the outer shell surface or internally. Internal contamination can be caused by the pathogen’s penetration through the eggshell or by direct contamination of egg contents, before oviposition, originating from infection of the reproductive organs [34]. In contrast, reptilian eggs are more permeable than avian eggs, due to their low calcium and high fibre contents. Furthermore, water uptake by the egg after it has been laid is crucial in the development of the reptilian embryo. For this reason, reptilian eggs are usually laid in locations with high humidity. Thus, both the permeable eggshell and the high humidity are factors that increase the likelihood of *Salmonella* penetrating the shell [35].

Indirect route of *Salmonella* spp. transmission from animals to humans is possible due to the ability of *Salmonella* cells to survive in the environment. One of these abilities is the biofilm production. *Salmonella* spp. can attach to many different spaces; they may be found e.g on vegetables, chicken eggs, and stainless steel or plastic [36–38]. Biofilm structures with cellulose and curli fimbriae as main components of the extracellular matrix promote the vertical transmission of *S. enterica* ser. Enteritidis in chicken [37]. *Salmonella* spp. was also found as a contamination of many surfaces near the animal living area, e.g. in vacuum cleaner bag, sink drain or on the door knob in household in which bearded dragon was kept, and in the kitchen, service area and public space of Antwerp Zoo [39,40].

Regardless of the route of *Salmonella* transmission in the environment, the faecal-oral route remains the most common that leads to human infection. It seems that the ingestion dose required to induce the infection depends on the *Salmonella* serovar, type and the way the food is handled, and the susceptibility of the host. Hara-Kudo *et al*. [41] indicated results for five different *Salmonella* serovars from eleven outbreaks in Japan [41]. From 7 outbreaks caused by *S. enterica* ser. Enteritidis, in two of them the infection rate was 100%. Based on their observation, it was determined that the ingested dose of this serovar was at least 3.51 × 10^6^ CFU (3.9×10^4^ per 1 g) but only 4 and 6 people were exposed to infection. In other outbreak caused by *Salmonella enterica* ser. Cerro, the minimal dose of pathogen was 1.6 × 10^3^ MPN (most probably number method) with 10% infection rate. The other analyzed *Salmonella enterica* serovars were: O4:H:eh,NT (ingested dose 2.6 × 10^6^ CFU, 7 × 10^2^ per 1 g), Montevideo (363 MPN, 66 and 960 MPN per 1 g) and Agona (<1500 CFU, <30 CFU per 1 g). *Salmonella enterica* ser. Montevideo and *Salmonella enterica* ser. Agona were the serovars with the lowest dose of pathogen needed to cause a disease after consuming salad with radish sprouts and fried soy pulp with egg, respectively [41].

During transmission to humans or animals, *Salmonella* spp. are faced with multiple stress factors such as a temperature, pH, salinity, metal ion stress, osmolarity, limiting nutrients and host immune defences. However, these pathogens are efficient enough to respond and adapt to these environments not only to survive but also to disseminate and retain its pathogenicity [42]. The ability of the bacteria to adapt to their host’s environment is regulated by many microbial features, which are responsible for the expression of clinical manifestations in specific host species. Host adaptation by *Salmonella* serovars occurs through two mechanisms: the acquisition of novel genetic elements encoding specific virulence factors, and loss of genes. Kisiela *et al*. [43] suggested that activation or inactivation of mannose-specific type 1 fimbrial adhesin FimH in different *Salmonella* serovars reflects their dynamic ability and course of adaptation to their specific host’s environment. The authors demonstrated that point mutations, horizontal gene transfer and genome degradation are responsible for differential pathoadaptive evolution of some *Salmonella* serovars [43]. Furthermore, *Salmonella* spp. can adapt to human hosts by changing their outer structures, such as lipopolysaccharide (LPS) and specific outer membrane proteins (OMPs). Those changes can lead to resistance of *Salmonella* spp. to human serum, especially the complement system which is part of the innate immune response [43–46]. *Salmonella* serovars isolated from reptiles are often resistant to human serum, which enables them to cause disease in humans. Strains isolated from the cloaca of the grass snake (*Natrix natrix*) from urban and touristic areas in Poland [30] have shown to be highly resistant to 50% human serum [data not published]. The possibility of carrying more than one *Salmonella* serovar by the same animal significantly increases the risk of genetic material exchange, which could lead to the acquisition of new virulence genes or other genetic factors.

## The role of diet in *Salmonella* spp. prevalence in pets – amphibians, reptiles, birds, rodents, cats and dogs

In spite of the widely discussed *Salmonella*-prevalence in animals, relatively little is known about the impact of diet that drives the possibility of the animal to become a carrier of this pathogen [47]. In this section, we reviewed the literature concerning the role of diet in *Salmonella*-prevalence in pets and showed risk factors associated with the increase of *Salmonella* spp. transmission from pets to humans.

Amphibians are carnivores and eat mostly earthworms, crickets, flies, moths, honeycomb moths, and small cockroaches, albeit bigger species of amphibians could be fed with small fish or mice [29]. *Salmonella* carrying in amphibians is generally asymptomatic. These pathogens are often isolated from frogs and toads than newts and salamanders, which can be the result of wider human contact with them in the environment (wild animals) or frequent breeding (pets). As amphibians obligatorily require access to water to live, contaminated water may become an indirect threat for humans. Additionally, amphibians are assumed to become carriers or suffer from a *Salmonella* spp. infection by consumption of contaminated feed or insects, which could be vectors of these bacteria, as described previously [48].

The diet of reptiles may be subjected to a variation depending on genera and species. *Salmonella*-prevalence in reptiles is reported to be higher in turtles than in lizards and snakes. In general, diets of omnivorous reptiles are usually balanced, containing both plant (herbs, crushed fruits, and vegetables) and animal components (mostly insects, young mice, snails). In contrast, herbivorous tortoises are characterized by a plant diet (herbs, fruits, and vegetables). Snakes and crocodiles are undoubtedly carnivores; in their diet animal feed is almost exclusive, consisting mainly of fish, chicks, rodents and other small mammals [33,49,50]. It is also important to note, that microbiota in the digestive tract of some reptiles may change periodically after long periods of fasting. Investigation of intestinal microbiota among herbivorous, omnivorous and carnivorous reptiles has shown that certain bacteria may become dominant, depending on diet, especially in animals kept in captivity. For instance, in herbivorous reptiles, highly varied gram-negative bacteria showed the highest prevalence, including *Salmonella* spp. Furthermore, Jiang *et al*. [51] observed the significant difference between the gut microbial community in loach-fed crocodile lizards than in the earthworm-fed and wild lizards. In addition, they found that the captive lizards fed loaches resulted in the enrichment of *Elizabethkingia, Halomonas, Morganella*, and *Salmonella spp*. Thus, this study proved that a diet promoting colonization of *Salmonella* spp. in the intestine of captive lizards may lead to the increased likelihood to transmit the pathogen from reptiles to humans [51]. The impact of diet in *Salmonella*-prevalence in captive reptiles were also reported in the US by Clancy *et al*. [52]. From a total of 175 samples isolated from 182 reptiles housed in Bronx Zoo, *Salmonella enterica* subsp. *enterica* was the most predominant (78/175; 45%). However, other non-enterica serovars were also identified, including *Salmonella enterica* subsp. *diarizonae* (42/175; 24%), many of which were clinically ill showing bony changes, dermatitis and anorexia. Authors determined that the strongest factors associated with an increased risk of illness in reptiles were carnivorous diet and prior confiscation [52].

**Figure 1.**
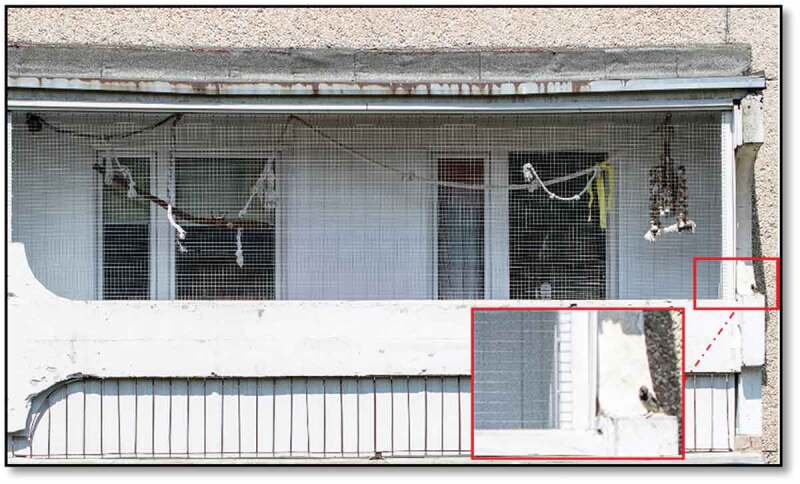
Home balcony keeping ornamental birds in Wałbrzych, Poland [author: Emil Paluch] (as the example of potential *Salmonella* spp. transmission from wild, free-living birds (zoom on the lower, right arrow) to ornamental birds outdoor)

Depending on the species, the diets of birds consist of animal and/or plant elements. Herbivorous birds forge on seeds, herbs, fruits, vegetables and special factory-made feed, while predatory birds such as eagles, hawks, falcons and owls are carnivores killing their prey by talons. Diet-dependent spreading of *Salmonella* spp. is associated with the contamination of feed by faeces, or in the case of omnivorous and carnivorous birds, with consumption of contaminated carrion, as well as colonized or ill prey [53]. The risk derives especially from the practice of releasing birds of prey during hunting. A similar risk occurs in outdoor pens of parrots or pigeons (i.e., kept on the balcony), these pens often have contact with wild, free-living birds, which can easily lead to contaminated feed (Figure 1). Much in this regard depends on the decisions of individual pet bird owners [51,53]. It is also worth bearing in mind that commercially available feeds used by pet owners may not provide sufficient nutrients, or consist of ill-balanced nutrients for a given species, leading to poor health and a higher susceptibility to infection or asymptotic carrying [53].

Young rodents, as all mammals, first thrive on their mother’s milk and upon reaching appropriate age become omnivorous. Their diets become very varied and contain many plant materials, as seeds, herbs, fruits, vegetables and animal components, as invertebrates, eggs or carrion [54]. Infected by *Salmonella* spp. rodents are involved in spreading the pathogen in their environment through faeces, which stay contagious up to three months. This may contaminate feed of other rodents, like fruits, vegetables, hay, fodder or water, and consequently lead to increased *Salmonella*-prevalence among other pet rodents [54,55].

Diet of cats and dogs is as varied as any omnivores; however, animal feed predominates, including raw meat [56]. Newborn cats and dogs consume their mother’s milk; however, dogs may also consume placenta and colostrum, which is beneficial for the formation of a healthy microbiota. Owners of older animals often introduce a diet of raw meat, including poultry [57]. According to a large, structured, 2016 survey in the US, 3% of dog and 4% of cat owners feed their pets in raw products, and raw or cooked human food was purchased for pets by 17% of dog owners [3]. Such a diet, despite its many benefits, carries a high risk of *Salmonella* spp. infection. For instance, Finley *et al*. [58] observed that when dogs are fed with *Salmonella*-contaminated food, they can become infected and consequently shed the bacteria in their faeces to contaminate the environment, other domestic animals, and even pet owners [58]. Experimental addition to dog’s diet probiotics containing *Lactobacillus* led to inhibition of *Salmonella* spp. growth. Probiotic lessened the gastrointestinal symptoms in ill dogs, albeit it also induced increased release of *Salmonella* to the environment, potentially leading to increased risk of infections in other animals. As Lowden *et al*. [9] have shown, commercial feed including dry food lowered the risk of asymptomatic carrying of *Salmonella* spp., but did not exclude it [9].

**Figure 2.**
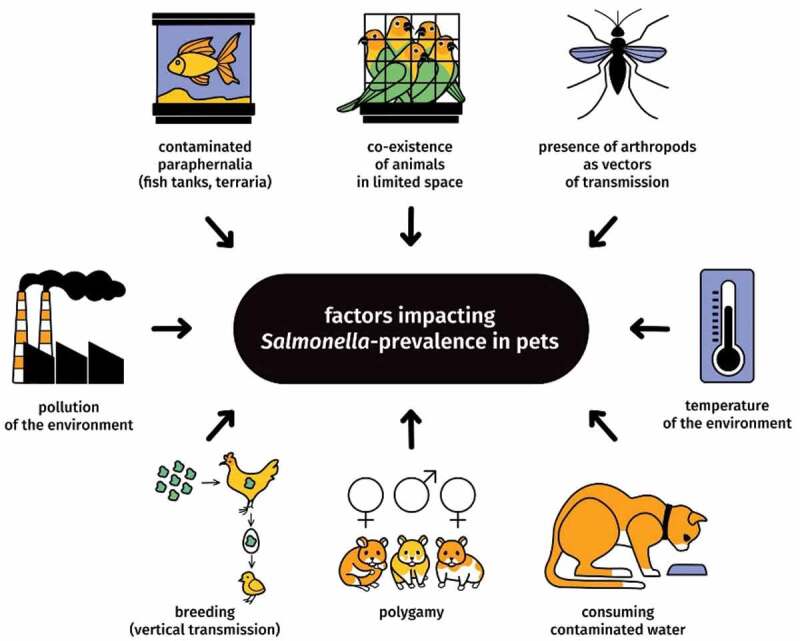
Factors influencing *Salmonella*-prevalence among domestic animals, exluding diet [29,35]

Other than diet, additional factors can influence *Salmonella*-prevalence among pets, including co-existence in limited space, environmental conditions, polygamy, presence of arthropods, contamination of paraphernalia, contact with wild animals, and others (Figure 2).

## *Salmonella* spp. prevalence in amphibians and reptiles

Amphibians and reptiles have become increasingly popular as pets worldwide. In the US alone, 4.5 million households own at least one reptile [3]. The most predominant are turtles, lizards and snakes. Nevertheless, up to 90% of reptiles are carriers of one or more *Salmonella* serovars [3,47]. In contrast, within the EU countries, less than 1% of human cases of salmonellosis are associated with exposure to reptiles [59]. Including amphibians kept at households, the most popular are frogs, salamanders and caecilians. In these animals, *Salmonella*-prevalence and associated cases of transmission to human are very limited compared to that of reptiles. However, their role is significant.

Since 2015, only a few reports determined amphibians as a source of human salmonellosis [46,60–62]. For instance, Ribas *et al*. [61] isolated 67 *Salmonella* strains from 97 frogs and toads (67/97, 69%) breed on Thailand farms and urban and protected areas; *Salmonella-*prevalence was 90%, 0% and 44.8%, respectively. The high *Salmonella*-prevalence in amphibians kept in farms (90%) confirms their significant role as vectors for the spread of salmonellosis to livestock. In this case, transmission to humans was considered as a result of indirect contact with amphibians. Of the eight identified in amphibians serovars, six of them (*S. enterica* ser. Hvittingfoss, *S. enterica* ser. Newport, *S. enterica* ser. Panama, *S. enterica* ser. Stanley, *S. enterica* ser. Thompson, and *S. enterica* ser. Wandsworth) led to human salmonellosis in Thailand. Farm-reared Chinese edible frogs (*H. rugulosus*) showed the highest *Salmonella-*prevalence (62%) [63]. In another study, Williams *et al*. [62] isolated 21 *Salmonella* serovars from 47 frogs (21/47, 45%). In this case, amphibian-associated salmonellosis appeared in 3 children keeping amphibians at households (3/15, 20%) [62]. These reports lead to the conclusion that awareness among amphibians’owners about potential risks of amphibian-associated salmonellosis is still required.

*Salmonella-*prevalence in free-living and captive reptiles in the period 2015–2021 was reported in the range from 2.1% (2020) [64] to 87.5% (2016) [30] in a global perspective. These studies were conducted in different countries, indicating the variety of *Salmonella* spp. geographic distribution in reptiles, regardless of climate or environment. Including European countries, studies in this area come from Croatia [65], Italy [63,66,67], Spain [68], Norway [31], Guadeloupe (French West Indies) [59], Poland [30,46,69,70], Portugal [71] and Slovenia [72] (Table 1). In Croatia, *Salmonella* spp. were detected in a total of 13% of the 200 healthy reptiles (including 31 lizards, 79 chelonians and 90 snakes) (26/200,13%). These vertebrates were kept as pets or housed in zoos [65]. In Italy, based on the faecal samples from 213 captive reptiles, 29 *Salmonella* isolates were detected (29/213,13.61%): 14 from 62 chelonians (14/62; 22.58%), 14 from 135 saurians (14/135; 10.37%), and 1 from 16 ophidians (1/16; 6.25%) [66]. In this country, *Salmonella* spp. were also detected in 3 of the 38 tortoises in a private breeding (3/38; 8%) and 15 turtles in the shelter (15/40; 37.5%) [63]. Also in Italy, Russo *et al*. [67] evaluated *Salmonella*-prevalence in housed gecko. Faecal swabs were collected from 70 apparently healthy captive gecko and *Salmonella* spp. were isolated from 24 of all samples (24/70; 34.3%) [67]. Furthermore, in Spanish Region (Valencia), Marin *et al*. [68] assessed *Salmonella* spp. carriage by pet reptiles in pet shops (54 reptiles) and households (69 reptiles). From all collected samples, 48% of pet reptiles carry *Salmonella* spp (59/123; 48%) [68]. In another study, after examination of faeces from 426 reptiles (322 anoles, 69 iguanas and 35 geckos) caught in Guadeloupe National Park, the frequency of *S. enterica* carriage was 15% (64/426, 15%) [59%]. In Slovenia, Romero *et al*. [72] examined the presence of 29.7% *Salmonella* spp. isolates from cloacal swabs of 74 reptiles (n = 22/76, 29.7%) kept at Ljubljana Zoo, Slovenia. The isolation prevalence was 38.6%, 18.2% and 12.5% in snakes, lizards and chelonians, respectively [72]. In Norway, 43% of the reptiles housed in three Norwegian zoos were shedding *Salmonella* spp., (44/103, 43%) with a prevalence of 62%, 67% and 3% in 53 snakes, 15 lizards and 35 chelonians, respectively [31]. In Poland, Nowakiewicz *et al*. [69] reported the presence of three serovars of *Salmonella enterica* (*S. enterica* ser: Newport, Braenderup and Daytona) in free-living European pond turtle (*Emys orbicularis*) with a low prevalence of 3% [69]. Furthermore, also in Poland, Dudek *et al*. [46] isolated 15 *Salmonella* spp. strains from 84 samples collected from reptiles housed in Wroclaw Zoo, Poland (15/84; 17.8%) [46]. Consistent to this study, Pawlak *et al.* [70] investigated cloacal Gram-negative microbiota of 45 free-living grass snakes (*Natrix natrix). Salmonella* spp. were present in 10 cloacal swabs (10/45, 22.2%) [70]. Moreover, also in grass snakes, Zając *et al*. [30] found *Salmonella* spp. in 14 from a total of 16 grass snakes (14/16, 87.5%) [30]. As of now, this study represents the highest prevalence of *Salmonella* in reptiles in Europe in 2015–2021.

**Table 1.** The distribution of *Salmonella* subspecies in reptiles in 2015–2021 (from the lowest to highest *Salmonella*-prevalence [%])

*Salmonella* prevalence [%]	Number of isolated serovars	Country of study	Publication year	Reptiles information	*Salmonella* subspecies prevalence [%]	Ref.
2.1%	15	Japan	2020	706 green anoles *(Anolis carolinensis)*	100% *enterica*	[64]
3%	3	Poland	2015	130 European pond turtles (*Emys orbicularis*)	100% *enterica*	[69]
4.19%	10	Canada	2018	236 Grand Cayman iguanas *(Cyclura lewisi)*	100% *enterica*	[80]
4.3%	4	Costa Rica	2015	115 Asian housed geckos (*H. frenatus*)	100% *enterica*	[73]
5%	2	New Zealand	2021	221 reptiles including 82 geckos and 139 sinks	100% *enterica*	[81]
5.2%	9	Malaysia	2017	171 snakes including boas, pythons and anacondas	Not showna	[75]
13%	14	Croatia	2015	200 reptiles including lizards, chelonians, and snakes	34.6% *enterica*, 23.1% *houtanae*, 23.1% *arizonae*, 15.4% *diarizonae*, 2.8% *salamae*	[65]
13.6%	29	Italy	2016	213 reptiles including chelonians, saurians and ophidians	89% *enterica* 11% *salamae*	[66]
15%	64	Guadeloupe	2019	426 reptiles including anoles, iguanas and geckos	73.8% *enterica* 26.2% *houtenae*	[59]
17.87%	15	Poland	2020	84 reptiles including lizards, agamas, anoles, tortoises and kingsnakes	53.3% *enterica* 26,7% *diarizonae* 20% *salamae*	[46]
18.9%	31	China	2016	164 pet turtles	100% *enterica*	[74]
22.2%	10	Poland	2020	45 grass snakes *Natrix natrix*	Not shown	[70]
29.7%	40	Slovenia	2016	74 reptiles including snakes, lizards and chelonians	63.6% *enterica* 31.8% *diarizonae* 4.5% *arizonae*	[72]
34.2%	26	Brazil	2019	76 reptiles, including lizards, chelonians, and snakes	50% *enterica* 34.6% *houtenae* 7.6% *diarizonae* 7.6% *arizonae*	[77]
34.3%	24	Italy	2018	70 geckos	83% *enterica* 8% *diarizonae* 8% *houtenae*	[67]
35.6%	16	Grenada	2020	45 Grenada bank tree boas *(Corallus grenadensis)*	100% *enterica*	[82]
41%	Not showna	Portugal	2021	78 reptiles including 43 turtles, 27 lizards and 8 snakes	40.6% *arizonae* 59.4% other subsp.	[71]
43%	44	Norway	2020	103 reptiles including snakes, lizards and chelonians	40% *enterica* 36% *diarizonae* 11% *salamae* 4% *arizonae* 2% *houtenae* 7% unknown	[31]
43.8%	20	Brazil	2020	153 black and white tegu lizards (*Salvator merianae*)	100% *enterica*	[76]
48%	59	Spain	2021	54 reptiles from pet shops and 69 reptiles from households	56.9% *enterica* 19.6% *houtenae* 11.8% *diarizonae* 9.8% *salamae* 2% *arizonae*	[68]
57%	57	Italy	2017	100 reptiles including snakes, lizards, turtles	Not showna	[14]
60%	36	USA	2015	60 reptiles including lizards, snakes, turtles and a combination of reptiles	88% *enterica* 12% other subsp.	[78]
83.3%	189	Japan	2019	227 small red-eared sliders *(Trachemys scripta elegans)*	57.3% *enterica* 29% other subsp.	[84]
87.5%	14	Poland	2016	16 snakes including 15 grass snakes *(Natrix natrix)* and 1 smooth snake *(Coronella austriaca)*	81.8% *diarizonae* 18.2% *enterica*	[30]

aIn these studies, the detected *Salmonella* isolates from reptiles were not differentiated for species, subspecies or serovars

Taking into account non-European countries and the period 2015–2021, *Salmonella*-prevalence in reptiles was reported in Costa Rica [73] China [74], Malaysia [75], Brazil [76,77], the US [52,78] Australia [79,80], New Zealand [81], Grenada [82], Canada [83] and Japan [12,64,84]. Relatively low *Salmonella*-prevalence was reported in 2020 in Japan. *Salmonella* strains, including *S. enterica* ser: Weltevreden and Enteritidis, were identified in samples extracted from 15 of the analyzed 706 free-living green anoles *(Anolis carolinensis*) (15/706; 2.1%) [64]. Including lizards, slightly increased *Salmonella*-prevalence was reported in Canada. Prud’homme *et al*. [83] collected 335 faecal samples from 236 captive and free-living Grand Cayman iguanas *(Cyclura lewisi). Salmonella*-prevalence ranged from 3.85% in iguanas housed in elevated wire-bottom enclosures to 6.06% in wild iguanas (the incidence of *S. enterica* in the population samples was 4.19%), demonstrating no significant difference among these conditions [83]. Furthermore, in Costa Rica, Jiminez *et al*. [73] examined faecal samples from 115 Asian house geckos (*Hemidactylus frenatus*) kept in houses and identified *Salmonella-*prevalence as 4.3% [73]. In contrast, Zhang *et al*. [74] found 31 *Salmonella* isolates from 164 faecal samples of pet turtles (31/164, 18.9%) kept in supermarkets and farmer’s markets in Shanghai, China [74]. *Salmonella* spp. were also isolated from 130 small red-eared sliders *(Trachemys scripta elegans)* retailed in pet shops in Japan, determining *Salmonella*-prevalence as 57.3% (130/227; 57.3%) [64]. Furthermore, Abba *et al*. [75] collected lung, liver, heart, kidney and intestine samples from the carcasses of snakes kept in two Malaysian zoos. *Salmonella-*prevalence in these reptiles ranged from 3.6% in pythons (5/139, 3.6%) to 33% (3/10, 33%) in boa [75]. Slightly increased *Salmonella*-prevalence was observed in free-living Grenada bank tree boas *(Corallus grenadensis)* in Grenada, with the number of 35.6% (16/45; 35.6%) [82]. Moreover, in Brazil, Ramos *et al*. [77] obtained faecal samples from 76 apparently healthy reptiles consisting of 15 lizards, 45 snakes and 16 chelonians. *Salmonella* spp. were isolated from 26 reptiles (26/76; 34.2%) [77]. Other Brazilian study has shown high prevalence of *S. enterica* serovars in black and white tegu lizard (*Salvator merianae*) which is an invasive species on the sampled area [76]. Increased *Salmonella*-prevalence was observed in Australia, where 52 from a total of 130 wild-caught reptiles were *Salmonella* spp. positive (52/130; 40%) [80]. In most of the introduced above studies, faecal samples were taken from captive reptiles kept in zoological gardens and pet shops or from free-ranging conditions. More intense interactions between reptiles and humans are in private holdings, so these percentages may be even higher than the general estimation. Furthermore, numerous factors carry widely between these studies, such as the source of *Salmonella* spp. isolation, diet, host’s environment, climate, antibiotic therapy, co-existence of other viral or parasitic diseases (Figure 2) as well as considerable variation in experimental design and the use of diagnostic techniques [31].

## Cases of reptile-associated salmonellosis in humans

The determination of the zoonotic potential of *Salmonella* spp. is important to highlight the problem of public health, particularly due to the increasing tendency of keeping such exotic animals as reptiles at households. For instance, in the US, of the 8389 non-typhoid salmonellosis case-patients, 290 (3.5%) reported reptile exposure. Including faecal samples of 60 reptiles, 36 (60%) yielded the same *Salmonella* serovar as the human isolate [78]. Krishnasamy *et al*. [85] described five *Salmonella* Paratyphi B variant L(+) tartrate + (Java) isolates in four American inhabitants keeping ball pythons (*Python regius)* as pets. The median patient age was 10 years (range ⩽1-40 years). No patient was hospitalized, and no deaths occurred [85]. In the US, the main outbreaks of human salmonellosis caused by turtle exposure occurred in 2015 and 2016. In 2015, based on the interview of 104 patients, 50 (48%) had contact with turtles. 18 (40%) of them were hospitalized, but no death occurred. The median age was 3 years (range <1–77 years). 21 positive *Salmonella* isolates were detected in turtles and 17 isolates matched the outbreak strains [85]. In 2016, a total of 133 patients with human salmonellosis were reported; 41% of them were children 5 years of age or younger. 55 (50%) of the 110 interviewed people reported contact with turtles or their environments; 38 patients were hospitalized, and no death was reported [86,87]. In Spain, Ricard *et al*. [88] reported a case of meningitis caused by *S. enterica* ser. Vitkin in a 1-month-old child after exposure to an aquatic pet turtle [88]. A very similar case was also reported in Spain. The same *Salmonella* serovar was isolated from the turtle faecal sample and blood of a two-year-old girl who had severe complications including high fever, sunken eyes and, pasty mucosa [89]. Furthermore, in Italy, Corrente *et al*. [13] conducted a cross-sectional study among reptile owners in order to assess a potential link between the presence of *Salmonella* spp. in their pets and the hygiene practices. From a total of 100 families, in 26 of them the potential risk of RAS occurred. Including 100 pet animals tested, *Salmonella*-prevalence was 57%. It was determined that co-habitation of the animals with other reptiles in the same terrarium was associated with a 2-fold increase in the risk of *Salmonella* spp infection. Animals handled by owners that did not report washing their hands after the cleaning procedures or the handling were exposed to a 3-fold increase in the risk of infection [13].

Considering reports from last 6 years and published by European scientists, reptile-associated salmonellosis with detection of the same *Salmonella* serovar in both patient’s blood and reptile faeces was observed in Switzerland – 2016 (years of publication) [90], UK (UK) – 2015 [91], Romania – 2017 [92], France – 2015 [88,93] and Spain – 2015 [88,89]. In Switzerland, the first case of reptile-associated sinusitis due to *S. enterica* subsp. *diarizonae* was reported in a 29-year-old snake handler who owned five pet snakes. In three snakes, the same *Salmonella* serovar was detected as in the blood of the owner. It was suggested that *Salmonella* spp. reached the upper respiratory tract hematogenously after oral inoculation or perhaps *via* inhalation [90]. In the UK, from 175 cases of human salmonellosis reported in the period 2010–2013, 48 patients had exposure to reptiles (48/175, 27.4%); 8 patients reported RAS salmonellosis with severe symptoms such as bacteraemia, meningitis and colitis requiring surgery. Almost half of RAS patients were hospitalized (23/48), but no deaths occurred [91]. Furthermore, in Romania, Gavrilovici *et al*. [92] reported a rare case of otitis with *Salmonella* spp. in a healthy 16-year-old adolescent, who was bathing in a village lake, where turtles were common. After taxonomic speciation, it turned out that the etiologic agent of this ear infection was *S. enterica* subsp *diarizonae*. Otitis was also associated with mastoiditis. Audiometric testing showed a moderately conductive hearing loss [92]. In France, the first isolation of *S. enterica* subsp. *arizonae* was reported in the bronchial aspirate from a patient suffering from pneumonia. The patient, a 73-year-old man kept snakes as pets [93].

Infants and children <5 years old are the most frequently exposed to RAS infections [11,14,78,89,94,95]. One study performed in Taiwan revealed that 31% of RAS cases occurred in children less than 5 years of age and 17% occurred in children aged 1 year or younger [94]. In other study, Kiebler *et al*. [96] investigated an outbreak of human salmonellosis in 133 people with exposure to pet bearded dragon lizards. The median patient age was 3 years (range, <1–79 years), 57% were aged ≤5 years, and 37% were aged ≤1 year [96]. Nevertheless, cases of RAS infection in adults and elderly people are also occurring, but in a lower frequency; mostly these infections are escorted with other, secondary infections. For instance, a 42-year-old patient from Equatorial Guinea experienced symptoms such as malaise, weakness, fever, and mild diarrhea. Based on the faecal sample analysis, *S. enterica* subsp. *salamae* was identified. During medical consultation, the patient reported regular consumption of sea turtle meat [97]. Furthermore, in Japan, Suzuki *et al*. [98] reported a case of pericarditis caused by *S. enterica* subsp. *arizonae* in a 36-year-old man with a history of type 2 diabetes mellitus. The patient was infected by pathogen transmission from pet snakes: a ball python *(Python regius)* and a Mexican black kingsnake *(Lampropeltis getula nigrita)* [98]. Nevertheless, these findings highlight the heightened risk in children and the potential for RAS to be transmitted without direct contact with the animal or its enclosure [99–101]. Furthermore, more hospitalizations occurred in RAS patients than non-RAS cases, suggesting that reptile-associated infection carries a higher likelihood of more severe symptoms with bloodstream infection [59].

The most commonly reported sources of RAS infection are *S. enterica* subsp. *salamae* (II), *S. enterica* subsp. *arizonae* (IIIb), *S. enterica* subsp. *diarizonae* (IIIb), *S. enterica* subsp. *houtenae* (IV) and *S. enterica* subsp. *indica* (VI). Including 2015–2021, *S. bongori* was not isolated from any of the samples collected from pets, so our analysis includes subspecies belonging to *S. enterica*. In one study, 73,124 human salmonellosis cases reported in the Netherlands over the last 30 years (1990–2020) were classified based on the source of infection. Of the total, 2% of cases were attributed to reptiles. The majority of *Salmonella* isolates (59%) belonged to *S. enterica* subsp. other than I, especially to *S. enterica* subsp. *diarizonae* (580/2281; 25.4%) [25]. This subspecies was also identified in 31.8% of reptiles housed in a zoo in Slovenia (24/74; 31.8%) [72], in 15.4% of reptiles housed in a zoo in Croatia (5/292; 15.4%) [65], in 13.3% of reptiles housed in a zoo in Poland (4/30; 13.3%) [46], in 36% of reptiles in three zoos in Norway (16/45; 36%) [31], in 7.6% of reptiles (6 species from a total of 76 free-living, captive and selected from private owner volunteers reptiles in Brazil [77], in 8% of geckos housed in private owners in Italy (6/70; 8%) [67], in 11.8% of reptiles housed in households and pet shops in Spain (41/349; 11.8%) [68] and in 81.8% of free-living snakes in Poland (13/16; 81.8%) [30]. *S. enterica* subsp. *diarizonae* was also detected in a Romanian 16-year-old adolescent, who was exposed to turtles [97] and in a 29-year-old Swiss snake handler [90]. Other *Salmonella* isolates from reptiles belonged to *S. enterica* subsp. *arizonae* (IIIa). This species was detected in 23.1% of reptiles kept in a zoo in Croatia (8/292; 23.1%) [65], in 4.5% of reptiles from a zoo in Slovenia (3/74;4.5%) [72], in 4% of reptiles housed in three zoos in Norway (2/45; 4%) [31], in 7.6% of reptiles (6 species from a total of 76 free-living, captive and selected from private owner volunteers reptiles in Brazil [77] and in 11.8% of reptiles selected from households and pet shops in Spain (41/349; 11.8%) [68]. *S. enterica* subsp. *arizonae* was also the etiologic agent of RAS case in a 1-month-old French child [88], a two-year-old Spanish child [89] and three children from the UK [91]. In all cases, infants had contact with exotic reptiles kept in households. Additionally, *S. enterica* subsp. *salamae* (II) was isolated in 10% of reptiles kept in a zoo in Poland (3/30;10%) [46], in 0.8% of wild and captive reptiles in Italy (2/213; 0.8%) [66], in 3.8% of reptiles kept in a zoo in Croatia (1/292; 3.8%) [65], in 9.8% of reptiles selected from households and pet shops in Spain (34/349; 9.8%) [68], and in 11% of reptiles housed in three zoos in Norway (5/45;11%) [31]. *S. enterica* subsp. *houteneae* (IV) was isolated in 23.1% of reptiles kept in a zoo in Croatia (8/292; 23.1%) [65], in 34, 6% of reptiles (26 from a total of 76 free-living, captive and selected from private owner volunteers reptiles in Brazil [77], in 8% of geckos housed in private owners in Italy (6/70; 8%) [67], in 2% of reptiles housed in three zoos in Norway (1/45;2%) [31], in 19.6% of reptiles selected from households and pet shops in Spain (68/349; 19.6%) [68] and in 26.2% wild reptiles in Guadeloupe (French West Indies) (17/65; 26.2%) [59]. Isolation of *Salmonella* serovars other than *S. enterica* subsp. *enterica* in reptiles is consistent with available literature reporting that clinical samples are more often associated with *S. enterica* subsp. *enterica* while other *Salmonella* subspecies correspond mainly to non-clinical samples and cause RAS infection with more severe complications in humans. All these results confirm, that reptiles serve nowadays as the main vector spreading non-commonly occurring *Salmonella* serovars into new ecological niches [46].

## *Salmonella* spp. prevalence in dogs

Dogs usually act as asymptomatic carriers of *Salmonella* spp; they are thought to shed one or more serovars intermittently for more than 6 weeks [102]. Rarely occurring clinical signs of salmonellosis in adult dogs and puppies include fever, loss of appetite, diarrhea, bloody diarrhea, abdominal pain, and abortion [103]. Other factors that may increase *Salmonella-*prevalence in dogs are the environment where animals live, contact with wild animals or other infected animals, differences in pet sanitary practices, feeding habits, public awareness about dog zoonosis, socioeconomic status of the owners, sample size, sampling strategies, and isolation methods performed [102,103].

Including 2015–2021, *Salmonella*-prevalence in household dogs was reported in different continents, indicating significant geographic variation in global perspective (Table 2). A study of 436 faecal samples from healthy dogs, including 126 samples from dogs kept in UK homes, reported *Salmonella* spp. only in one female terrier breed (1/4366, 0.23%) [9]. In another study, from a total of 325 healthy dogs across Spain, *Salmonella-*prevalence was 1.85% (6/325, 1.85%) [103]. Furthermore, Reimschuessel *et al*. described that 60 diarrheic and non-diarrheic dogs from a total of 2422 dog population were *Salmonella*-positive (60/2422, 2.5%). Faecal samples were solicited from different geographically dispersed veterinary laboratories in the US. This study confirmed statistically higher prevalence in diarrheic dogs (3.8%) than in non-diarrheic dogs (1.8%) [104], which is in concordance with other reports [8,105–107]. Faecal samples collected from 144 non-diarrhoeic dogs in Grenada revealed that 5.6% (8/144) of them were *Salmonella* positive [105]. A similar percentage was also observed in Western Australia. Of the 405 faecal samples obtained from dogs placed from the different environment: animal shelters, racing greyhounds or households, 5.4% were *Salmonella*-positive (22/405, 5.4%) [108]. A slightly higher percentage of *Salmonella-*prevalence in 243 dogs was observed in China (23/243, 9.47%) [28]. Furthermore, investigations undertaken in Ethiopia and Equador represent even higher prevalences, with the percentage of 11.7% [109] and 12.5% [110], respectively. The goal of Wu *et al*. [106] study was to investigate the association between *Salmonella* spp. infection, pet dogs and their caregivers in Thailand. *Salmonella*-prevalence was observed in 18 companion dogs from a total of 140 analyzed (18/140, 12.86%) [106]. As conclusion, dogs may be potential agents of salmonellosis, especially when multiple different factors (e.g. weakened immune system, improper diet, rich in raw food, and indecent environmental and animal welfare commitments) contribute to the increased risk of pathogen transmission to dog owners.

**Table 2.** *Salmonella*-prevalence in dogs in 2015–2021 (from the lowest to highest *Salmonella* prevalence [%])

*Salmonella* prevalence [%]	Number of *Salmonella*-positive dogs	Country	Publication year	Number of tested dogs	*Salmonella* serovar/s or subspecies	Ref.
0.23%	1	UK	2015	436	**1 isolate**a**: *Salmonella enterica* subsp**. *arizonae* (1/1, 100%)	[10]
1.85%	6	Spain	2020	325	**3 *Salmonella enterica* serovars**: Havana (3/325) *S*. Mikawasima (2/325) *S*. monophasic Typhimurium (1/325)	[103]
2.5%	60	US	2017	2422	**24 *Salmonella enterica* serovars, 64 isolates** the most predominant: *S*. Newport (13/64, 20.3%), *S*. Enteritidis (5/64, 7.8%), *S*. Javiana (5/64, 7.8%), *S*. Infantis (5/64, 7.8%), *S*. Typhimurium (4/64, 6.35%),	[104]
4,9%	27	US	2015	554b	**10 *Salmonella enterica* serovars, 27 isolates** the most predominant: *S*. Newport (6/27, 22%) *S*. Javiana (4/27,15%) *S*. Braenderup (2/27, 7%) *S*. Infantis (2/27, 7%)	[102]
5.4%	22	Australia	2019	405	**Not specified**	[107]
5.6%	8	Grenada, West Indies	2018	144	**6 *Salmonella enterica* serovars, 35 isolates** *S*. Arechavaleta (13/35, 37.1%) *S*. Montevideo (5/35, 14.3%) *S*. Javiana (2/35, 5.7%) *S*. Rubislaw (5/35, 14.3%) *S*. Braenderup (5/35, 14.3%) *S*. Kiambu (5/35, 14.3%)	[105]
6.27%	24	Mexico	2019	385	**24 *Salmonella* isolates**a *S. enterica* subsp. *enterica* (21/24, 87.5%) *S. enterica* subsp. *arizonae* (3/24, 12.5%)	[111]
9.47%	25	China	2020	243	**8 *Salmonella enterica* serovars** *S*. Kentucky (11/25, 44%), *S*. Indiana (5/25,20%), *S*. Typhimurium (4/25,16%) *S*. Derby (1/25, 4%) *S*. Toucra (1/25, 4%) *S*. San Diego (1/25, 4%) *S*. Newport (1/25, 4%) *S*. Saint Paul (1/25, 4%)	[28]
11%	11	Iran	2018	100b	*S. enterica* ser. Typhimurium (7/11, 63.4%) *S. enterica* ser. Enteritidis 36.4%)	[112]
11.7%	42	Ethiopia	2017	360	**14 *Salmonella enterica* serovars** the most predominant: *S*. Bronx (7/42, 16.7%), *S*. Newport (6/42, 14.3%), *S*. Typhimurium (4/42, 9.5%), *S*. Indiana (4/42, 9.5%), *S*. Kentucky (4/42, 9.5%), *S*. Saint Paul (4/42, 9.5%) *S*. Virchow (4/42, 9.5%)	[109]
12.5%	5	Equador	2016	267	*S*. ***enterica*** ser. Infantis (5/267, 1.9%)	[110]
12,86%	18	Thailand	2020	140	**13 *Salmonella enterica* serovars** the most predominant: *S*. Stanley (3/18, 16.67%) *S*. Hvittingfoss (3/18, 16.67%) *S. enterica* serotype I 1,4, [5],12:i:- (2/18, 11.20%)	[106]

aThese studies did not include the *Salmonella* subsp. differentiation into serovars

bFaecal samples were obtained from shelter dogs

*Salmonella* prevalence in dogs is also highly variable depending on the environment in which the animals live. For instance, *Salmonella* isolation rates from stray dogs and shelter dogs are higher than those from household dogs. This phenomenon may be due to the increased freedom to roam and scavenge, possible close contact with carcasses or offals of wildlife and raw and undercooked food [103]. In Spain, Bataller *et al*. [103] obtained 1 *Salmonella*-positive rectal swab from 85 dogs kept in households (1.17%) and 3 *Salmonella*-positive samples from 84 dogs kept in animal shelters (3.57%) [103]. Furthermore, in Texas, US, *Salmonella* prevalence from shelter dogs was 4.9% (27/554) [102]. In Mexico, Castro *et al*. [111] identified 6.27% of *Salmonella* spp. isolated from 385 stray dogs in urban, rural and coastal areas (24/385) (no significant statistical differences were detected in different geographical regions) [111]. Moreover, in Iran, a total of 100 faecal swabs and blood samples were obtained from symptomatic and apparently healthy shelter dogs; 11 samples (11%) of them were *Salmonella*-positive [112]. These observations indicate the serious problem of public health especially in urban communities, where a massive population of stray dogs in cities exists with no certain monitoring and control system over their nutritional habits, potentially leading, in consequence, to transmission of *Salmonella* spp. to humans [112,113].

## *Salmonella* spp. prevalence in cats

Several reports published in 2015–2021 have concluded that contact with healthy cats kept in homes does not constitute a major zoonotic risk of salmonellosis. Only a few cases were reported, in which salmonellosis was passed on from cats to humans [27,28,104,114,115]. For instance, in China, Wei *et al*. [28] collected faecal samples from 113 cats and only two cats (with and without diarrhoea) were *Salmonella*-positive (2/113, 1.77%) [28]. In Western Australia, Aeh and Stayt [108]. reported the prevalence of faecal pathogens in the microbiome of cats with diarrhoea. Of 289 feline faecal samples reviewed, *Salmonella* spp. (1.7%) were detected, mostly in young cats (range 14 weeks to 2 years and 10 months) [108]. Interestingly, Vercelli *et al*. [116] detected *S. enterica* ser. Typhimurium in the urine culture of a cat suffering from endocarditis and myocarditis [116]. Introduced above reports show a relatively low *Salmonella*-prevalence in cats.

## *Salmonella* spp. prevalence in ornamental birds

Among birds most often kept by humans are parrots, canaries, European goldfinches, pigeons, and increasingly popular birds of prey like owls and falcons. However, including 2015–2021, a low frequency of *Salmonella-*prevalence was reported in ornamental birds kept in households. Most of the studies relate to *Salmonella* spp. transmission to humans by indirect contact of pet birds with other companion or wild animals. For instance, when pet birds are gathered in an exhibition in open-air aviaries, other animals having access to these places come in direct contact with them (like in the example shown in Figure 1). This contact may be a source of indirect *Salmonella* spp. carriage to humans, especially including the fact that *Salmonella* can survive for extended periods on wood and dust and can live for 28 months in avian faeces [117]. In one study, it was determined that domestic cats and dogs were linked to *Salmonella* spp. transmission from wild birds (81% and 52% of cat and dog cat isolates, respectively, shared a common *Salmonella* serovar with birds) [118]. Furthermore, Mather *et al*. [119] determined that some subtypes of *S. enterica* ser. Typhimurium – definitive phage types (DTs) 40, 56 variant and 160 – are host-adapted to wild passerine birds (e.g. finches, sparrows), and these birds may represent a reservoir of infection for humans and other companion animals, especially those kept outdoor (for example as shown in Figure 1 or dogs/cats partly allowed to roam outdoor) [120,119]. Moreover, de Oliviera *et al*. [119,120] obtained cloacal swabs from 156 free-ranging urban birds including synanthropic great egrets (*Ardea alba*) and feral pigeons (*Columba domestica*) that inhabited the surroundings of an urban zoo in Brazil to assess shelter and food. By defecating in these areas, they potentially contribute to the *Salmonella*- transmission to the captive in zoo animals. A total of 11 birds were positive for *S. enterica* ser. Typhimurium (11/156; 7%) showing that these free-ranging birds are possible sources of infection to other animals [119,120]. In urban infrastructure, synanthropic birds such as domestic pigeons, house sparrows or common chaffinches find abundant food and places for roosting and nesting. This phenomenon may create opportunities for frequent contact with humans and other animals. Pigeon dropping may be a potential risk of *Salmonella* spp. transmission through contamination of drinking water sources or agricultural crops [121]. Sharing the same environmental condition where outdoor pets have contact with pigeon droppings may lead to *Salmonella* spp. passage and, in that way, pets become asymptomatic carriers of this pathogen. In conclusion, although cases of *Salmonella*-transmission from pet birds to humans are rare, caution should still be exercised when engaging in contact with these companion animals. Furthermore, limiting the contact between wild birds and pet birds and their foods is another valid measure to prevent unnecessary transmission.

## *Salmonella* spp. prevalence in rodents

Due to their small sizes and relatively low purchase and maintenance costs, rodents (e.g. hamsters, rats, mice, gerbils and guinea pigs) did not lose their popularity as pets in recent years. However, including 2015–2021, more cases of *Salmonella* spp. transmission to humans were associated with wild rodents rather than their captive counterparts [122]. In one study, Himsworth *et al*. [123] detected *Salmonella* spp. in 3/633 (0.5%) Norway and black rats (*Rattus norvegicus* and *Rattus rattus*, respectively) from an urban neighborhood of Vancouver, Canada. The most predominant were *S. enterica* ser. Derby, *S. enterica* ser. Indiana and *S. enterica* ser. Enteritidis. It was suggested that rats acquired *Salmonella* spp. from their environment [123]. Furthermore, the recently published study aimed to estimate the prevalence of diarrheagenic *Escherichia coli* (DEC) and *Salmonella* spp. in urban slum environments in Brazil. *S. enterica* was found in only one (1.4%) of 67 brown rats (*R. norvegicus*) [124]. Altogether, including the fact that since 2015 we did not find the literature detecting cases of *Salmonella* spp. in pet rodents and a low number of articles determined *Salmonella* spp. exposure in wild rodents, we are inclined to ascertain that there is a significantly low possibility to be *Salmonella*-infected by contact with these animals. However, these cases may occur and should not be omitted.

Nevertheless, considering rodents individually, guinea pigs are highly susceptible to *Salmonella* spp. and hence, they need more attention. These animals are the most frequently kept as pet rodents, with 0.8 million in the UK and 1.36 million in the US in 2019 [125]. They are often selected as pets due to their placid, docile temperament and ease of handling [54]. However, *Salmonella-*infected guinea pigs exhibit reduced physical activity, social interaction progressing, lethargy, and anorexia. Reduced physical activity can induce gut stasis which can cause rapid deterioration resulting in sudden death [57]. The incubation period is 5–7 days [58]. Aging, other diseases, malnutrition, and environmental stress are predisposing factors to develop severe clinical symptoms of salmonellosis in guinea pigs [57].

Due to the high susceptibility to *Salmonella* spp., guinea pigs are thought to become carriers, which in turn make them a potential source of *Salmonella* spp. transmission to humans. For instance, in 2017, two *S. enterica* ser. Enteritidis isolates were detected in 9 American inhabitants who reported exposure to pet guinea pigs, which were purchased from two pet stores. Five *Salmonella* isolates from guinea pigs matched the outbreak strain. The median patient age was 12 years (range = 1–70 years). One patient was hospitalized, and no deaths were reported [126–131]. In conclusion, guinea pigs may act as potential sources of human salmonellosis caused by direct or indirect contact with humans. However, it is worth bearing in mind that household guinea pigs as rodents are not likely to be a source of human salmonellosis, even if they are highly susceptible to be *Salmonella* spp.

## The importance of wildlife trade

International importations of free-living animals are one of the major drivers of salmonellosis emergence and results in its globalization. Illegal wildlife trade (for example, for companion or ornamental pets), is the world’s fourth largest illegal business after narcotics, counterfeiting and human trafficking [132]. Although the scale of the illegal market is unknown, it was calculated that approximately 5.9–9.8 million reptiles were (legally) imported to the EU in 2009 alone, a substantial rise from the 1.6 million imported in 2005 [133]. Including European countries, Germany is by far the largest importer of live reptiles within the EU. In this country, 1532 valid reptile species and 352 valid amphibian species had been recorded in the German pet trade in 2017–2018 [134]. Another report showed that, from 2013 to 2014, about 490,750 exotic individual animals were legally imported to the Netherlands. 43% of them were destined for the Netherlands, a small number (4%) was destined for other EU countries and the rest (53%) were in transit to other non-European countries. The majority of the animals imported in the Netherlands were reptiles (93.8%), followed by amphibians (5.8%), birds (0.06%) and mammals (0.4%). The animals originated predominantly from the US (78.8%), Vietnam (5.1%), Indonesia (3.5%) and Tanzania (3.1%) [134]. Furthermore, Green *et al.* [135] evaluated the trade in live wild animals entering the UK in 2014–2018 using data reported by the Animal and Plant Health Agency (APHA). Over 8 million individual animals were imported into the UK from 90 countries across nine global regions. Amphibians were the most commonly imported group (73%), followed by reptiles (17%), mammals (4%), and birds (3%). The highest number of import records came from Europe and Africa, but the largest volume of animals came from North America and Asia [135]. Since exotic amphibians and reptiles are not tested for *Salmonella* spp. and a large number of them are imported by trade companies (99.8%) and mostly destined for the pet industry, the probability of exposure of humans to *Salmonella* spp. is high [134]. The scale of international trade is likely to be even greater than current estimates due to incomplete record-keeping and widespread illegal activity throughout the industry [135]. Thus, due to observed more interactions with humans by international trade of free-living amphibians and reptiles and human urbanization resulting in increasing human encroachment into natural ecosystems, the role of these animals in *Salmonella* distribution is incontrovertible [135]. A special field where a wildlife trade takes place are wet markets. These types of markets are especially popular among low-income communities of Asia, Africa and Latin America. While countries have drawn the attention to wet markets due to COVID-19 pandemic, these areas can be also an important sources of other zoonoses such as salmonellosis [136]. Factors predisposing *Salmonella* spp. occurrence in wet markets are poor animal keeping conditions (overcrowding, cramped cages, transport mortality, wrong or insufficient food, proximity to other animal and species, stress, injuries and diseases), poor sanitation (lack of toilets and hand washing stations) and the possibility to contaminate fresh food and meat by shedding bacteria from wildlife animals [137,138]. To date, a lot of studies relied on the contamination of different types of meat including chicken, beef and pork by *Salmonella enterica* in China, Philippines, Malaysia and Vietnam [139–141]. The studies from Asia also confirmed presence of virulence genes and multi drug resistant and ESBL producing (*extended-spectrum beta-lactamases*) fenotypes of *S. enterica* isolated from meat sampled in wet markets [142,143]

## Pet regulations and guidelines for pet owners

Animal-human relationships may reduce human stress and ailments. However, these interactions may also have harmful effects, including the spread of salmonellosis. A study conducted among 401 Canadian pet owners revealed a range of practices that increase *Salmonella*-disease risk, for instance: allowing dogs and cats to sleep in a child’s bed, allowing dogs to lick a child’s face, and allowing a reptile to roam through the kitchen. Although the hand washing by children was high (76% washed hands after touching the pet, its feces or housing), the authors concluded there is still a high need to educate people on *Salmonella*-disease-prevention practices [144]. Different national and international organizations, including the World Health Organization (WHO), CDC, the Association of Reptilian and Amphibian Veterinarians (ARAV) and the American Pet Products Association (APPA) are providing pet owners in the recommendation on how to prevent or at least minimize salmonellosis well as to promote and develop responsible pet ownership and the pet products industry [145]. These organizations support and monitor the industry legislations and regulations. Although *Salmonella* occurs globally, these pathogens are most commonly detected in areas, where intensive animal husbandry is practiced. In some countries, *Salmonella* infections were eliminated in domestic animals due to *Salmonella* eradication programs. In Sweden, according to the Swedish law on zoonoses (Zoonoslagen, SFS 2006∶1039), every case of *Salmonella* spp. isolation from domestic animal, animal product or feed should be reported and measures to eradicate *Salmonella* should be taken at any positive finding [145].

## Conclusion

Bacteria *Salmonella* spp. are still one of the most serious global problems of public health affecting approximately 1.3 billion cases of illness every year. To date, several different routes of *Salmonella* spp. transmission to human were reported, both indirect (for example by environment) and direct (by consumption of contaminated food or close contact with infected animals). Due to increasing frequency of keeping exotic animals like amphibians, reptiles and ornamental birds at households, their role in the transmission of *Salmonella* spp is growing. Based on the current literature regarding *Salmonella* spp. isolation and characterization in pets, we indicated bacterial zoonotic potential of pet-to-human transmission. It is worth noting that *Salmonella*-prevalence in pets depends on many aspects including diet, co-existence with other animals in limited space, environmental conditions, potential contact with wild animals and others. Based on collected data of *Salmonella*-prevalence in pets, we emphasize that when considering adopting and keeping companion animals, it is important to be aware of potential routes of *Salmonella* spp. transmission and their consequences of human health.

## Data Availability

The authors confirm that the data supporting the finding of this study are available within the article (http://dx.doi.org/10.1080/20008686.2021.1975530).

## References

[1] Amiot C, Bastian B, Martens P. People and companion animals: it takes two to tango. BioScience. 2016;66(7):552–19.

[2] Pet Insurance Reviews, Pet Statistics, PetInsuranceReviews.org, 2015 (accessed on 2021 Apr 25).

[3] 2019-2020APPA National Pet Owners Survey. In: Pet Industry Market Size & Ownership Statistics. 2019-2020. https://www.americanpetproducts.org/pubs_survey.asp, (accessed on 2021 Apr 25).

[4] The European Pet Food Industry, European Statistics, 2021. https://fediaf.org/who-we-are/european-statistics.html. 2021 Aug 20

[5] Stull JW, Brophy J, Weese JS. Reducing the risk of pet-associated zoonotic infections. CMAJ. 2015;187(10):736–743. 25897046 10.1503/cmaj.141020 PMC4500695

[6] Ebani VV. Domestic reptiles as source of zoonotic bacteria: a mini review. Asian Pac J Trop Med. 2017;10(8):723–728. 10.1016/j.apjtm.2017.07.020 28942820

[7] Moretti A, Agnetti F, Mancianti F, et al. Dermatophytosis in animals: epidemiological, clinical and zoonotic aspects. G Ital Di Dermatologia E Venereol. 2013;148(6):563–572. 24442037

[8] Pereira A, Martins A, Brancal H, et al. Parasitic zoonoses associated with dogs and cats: a survey of Portuguese pet owners’ awareness and deworming practices. Parasites Vectors. 2016;9(1). DOI:10.1186/s13071-016-1533-2. PMC4862121 27160667

[9] Lowden P, Wallis C, Gee N, et al. Investigating the prevalence of *Salmonella* in dogs within the Midlands region of the UK. BMC Vet Res. 2015;11(1). DOI:10.1186/s12917-015-0553-z. PMC4574458 26381479

[10] Scheelings TF, Lightfoot D, Holz P. Prevalence of *Salmonella* in Australian reptiles. J Wildl Dis. 2011;47(1):1–11. 21269991 10.7589/0090-3558-47.1.1

[11] Nakadai A, Kuroki T, Kato Y, et al. Prevalence of *Salmonella* spp. in Pet Reptiles in Japan. Pet Reptiles in Japan. J Vet Med Sci 2005;67(1):97–101. 10.1292/jvms.67.97 15699603

[12] Ebani VV, Cerri D, Fratini F, et al. *Salmonella enterica* isolates from faeces of domestic reptiles and a study of their antimicrobial in vitro sensitivity. Res Vet Sci. 2005;78(2):117–121. 15563917 10.1016/j.rvsc.2004.08.002

[13] Corrente M, Madio A, Friedrich KG, et al. Isolation of *Salmonella* strains from reptile faeces and comparison of different culture media. J Appl Microbiol. 2004;96(4):709–715. 15012809 10.1111/j.1365-2672.2004.02186.x

[14] Rabsch W, Plenz B, Fruth A, et al. Evidence for the transmission of *Salmonella* from reptiles to children in Germany, July 2010 to October 2011. Euro Surveill. 2013;18(46):20634. 24256890 10.2807/1560-7917.es2013.18.46.20634

[15] Kurtz JR, Goggins JA, McLachlan JB. *Salmonella* infection: interplay between the bacteria and host immune system. Immunol Lett. 2017;190:42–50. 28720334 10.1016/j.imlet.2017.07.006 PMC5918639

[16] The European Union One Health 2018 Zoonoses Report. In: European Food Safety Authority and European Centre for Disease Prevention and Control (EFSA and ECDC). EFSA J. 2019;17(12):e05926, https://efsa.onlinelibrary.wiley.com/doi/10.2903/j.efsa.2019.5926(accessed on 25 Aug 2021). 10.2903/j.efsa.2019.5926 PMC7055727 32626211

[17] Porwollik S, Boyd EF, Choy C, et al. Characterization of *Salmonella enterica* Subspecies I Genovars by Use of Microarrays. J Bacteriol. 2004;186(17):5883–5898. 10.1128/JB.186.17.5883-5898.2004 PMC516822 15317794

[18] Issenhuth-Jeanjean S, Roggentin P, Mikoleit M, et al. Supplement 2008-2010 (no. 48) to the White-Kauffmann-Le Minor scheme. Res Microbiol. 2014;165(7):526–530. 25049166 10.1016/j.resmic.2014.07.004

[19] Lamas A, Miranda JM, Regal P, et al. A comprehensive review of non-entericasubspecies of *Salmonella enterica*. Microbiol Res. 2018;206:60–73. 29146261 10.1016/j.micres.2017.09.010

[20] Gal-Mor O. Persistent infection and long-term carriage of typhoidal and nontyphoidal salmonellae. Clin Microbiol Rev. 2019;32(1):e00088–18. 30487167 10.1128/CMR.00088-18 PMC6302356

[21] Gal-Mor O, Boyle EC, Grassl GA. Same species, different diseases: how and why typhoidal and non-typhoidal *Salmonella enterica* serovars differ. Front Microbiol. 2014;5:391. 25136336 10.3389/fmicb.2014.00391 PMC4120697

[22] GBD 2016 Disease and Injury Incidence and Prevalence Collaborators. Global, regional, and national incidence, prevalence, and years lived with disability for 328 diseases and injuries for 195 countries, 1990-2016: a systematic analysis for the Global Burden of Disease Study 2016. Lancet. 2017;390(10100):1211–1259. 10.1016/S0140-6736(17)32154-2 PMC5605509 28919117

[23] Guarino A, Ashenazi S, Gendrel D, et al. European Society for Pediatric Gastroenterology, Hepatology, and Nutrition/European Society for Pediatric Infectious Diseases evidence-based guidelines for the management of acute gastroenteritis in children in Europe: update 2014. J Pediatr Gastroenterol Nutr. 2014;59(1):132–152. 24739189 10.1097/MPG.0000000000000375

[24] Mughini-Gras L, Heck M, Van Pelt W Increase in reptile-associated human salmonellosis and shift toward adulthood in the age groups at risk, the Netherlands, 1985 to 2014. Euro Surveill. 2016;21(34):30324. DOI: 10.2807/1560-7917.ES.2016.21.34.30324. PMC5144934 27589037

[25] Fardsanei F, Dallal MMS, Douraghi M, et al. Genetic diversity and virulence genes of *Salmonella enterica* subspecies *enterica* serotype Enteritidis isolated from meats and eggs, Microb. Pathog. 2017. DOI:10.1016/j.micpath.2017.04.026 28433796

[26] Demirbilek SK. Salmonellosis in Animals. In: Mascellino, M.T., Ed.;Salmonella - A Re-emerging Pathogen. ; IntechOpen: Budapest, Hungary, 2017. p.18. DOI: 10.5772/intechopen.72192.

[27] Wei L, Yang C, Shao W, et al. Prevalence and drug resistance of *Salmonella* in dogs and cats in Xuzhou. China J Vet Res. 2020;64(2):263–268. 32587913 10.2478/jvetres-2020-0032 PMC7305642

[28] Hoelzer K, Switt A, Switt M. Animal contact as a source of human non-typhoidal salmonellosis-Review. Vet Res. 2011;42(1):34. 21324103 10.1186/1297-9716-42-34 PMC3052180

[29] Ahmer BM, Gunn JS. Interaction of Salmonella spp. with the Intestinal Microbiota. Front Microbiol. 2011;2(101). DOI:10.3389/fmicb.2011.00101. PMC3131049 21772831

[30] Zając M, Wasyl D, Różycki M, et al. Free-living snakes as a source and possible vector of *Salmonella* spp. and parasites. Eur J Wildl Res. 2016;62(2):161–166.

[31] Bjelland AM, Sandvik LM, Skarstein MM, et al. Prevalence of *Salmonella* serovars isolated from reptiles in Norwegian zoos. Acta Vet Scand. 2020;62(1). DOI:10.1186/s13028-020-0502-0 PMC6953243 31918736

[32] De Lucia A, Rabie A, Smith SR, et al. Role of wild birds and environmental contamination in the epidemiology of *Salmonella* infection in an outdoor pig farm. Vet Microbiol. 2018;227(227):148–154. 30473346 10.1016/j.vetmic.2018.11.003

[33] Xu Y, Tao S, Hinkle N, et al. *Salmonella*, including antibiotic-resistant *Salmonell*a, from flies captured from cattle farms in Georgia, U.S.A. Sci Total Environ. 2018;616-617:90–96. 29107782 10.1016/j.scitotenv.2017.10.324

[34] Gantois I, Ducatelle R, Pasmans F, et al. Mechanisms of egg contamination by *Salmonella Enteritidis*. FEMS Microbiol Rev. 2009;33(4):718–738. 19207743 10.1111/j.1574-6976.2008.00161.x

[35] Gilbert MJ, Duim B, Zomer AL, et al. Living in cold blood: *arcobacter, Campylobacter*, and *Helicobacter* in reptiles. Front Microbiol. 2019;10:1086. 31191467 10.3389/fmicb.2019.01086 PMC6530492

[36] Lee KH, Lee JY, Roy PK, et al. Viability of *Salmonella* Typhimurium biofilms on major food-contact surfaces and eggshell treated during 35 days with and without water storage at room temperature. Poult Sci. 2020 Sep;99(9):4558–4565. 32868000 10.1016/j.psj.2020.05.055 PMC7598110

[37] Chen S, Feng Z, Sun H, et al. Biofilm-Formation-Related Genes csgD and bcsA Promote the Vertical Transmission of *Salmonella Enteritidis* in Chicken. Front Vet Sci. 2021;7(625049):Published 2021. DOI:10.3389/fvets.2020.625049. PMC7840958 33521095

[38] Elpers L, Kretzschmar J, Nuccio SP, et al. Factors Required for Adhesion of *Salmonella enterica* Serovar Typhimurium to Corn Salad (*Valerianella locusta*). Appl Environ Microbiol. 2020;86(8):e02757–19. 32033951 10.1128/AEM.02757-19 PMC7117930

[39] Lowther SA, Medus C, Scheftel J, et al. Foodborne Outbreak of *Salmonella* Subspecies IV Infections Associated with Contamination from Bearded Dragons. Zoonoses Public Health. 2011;58(8):560–566. 21824356 10.1111/j.1863-2378.2011.01403.x

[40] Bauwens L, Vercammen F, Bertrand S, et al. Isolation of *Salmonella* from environmental samples collected in the reptile department of Antwerp Zoo using different selective methods. J Appl Microbiol. 2006;101(2):284–289. 16882135 10.1111/j.1365-2672.2006.02977.x

[41] Hara-kudo Y, Takatori K. Contamination level and ingestion dose of foodborne pathogens associated with infections. Epidemiol Infect. 2011;139(10):1505–1510. 21205441 10.1017/S095026881000292X

[42] Pradhan D, Negi VD. Stress-induced adaptations in *Salmonella*: a ground for shaping its pathogenesis. Microbiol Res. 2019;229:126311. 31446332 10.1016/j.micres.2019.126311

[43] Kisiela DI, Chattopadhyay S, Libby SJ, et al. Evolution of *Salmonella enterica* Virulence via point mutations in the fimbrial adhesin. PLoS Pathog. 2012;8(6):e1002733. 10.1371/journal.ppat.1002733 PMC3369946 22685400

[44] Bugla-Płoskońska G, Rybka J, Futoma-Kołoch B, et al. Sialic Acid-Containing Lipopolysaccharides of *Salmonella* O48 Strains—Potential Role in Camouflage and Susceptibility to the Bactericidal Effect of Normal Human Serum. Microb Ecol. 2010;59(3):601–613. 10.1007/s00248-009-9600-2 19844648

[45] Pawlak A, Rybka J, Dudek B, et al. *Salmonella* O48 serum resistance is connected with the elongation of the lipopolysaccharide O-antigen containing sialic acid. Int. J. Mol. Sci. 2017;18(10):2022. 10.3390/ijms18102022 PMC5666704 28934165

[46] Dudek B, Książczyk M, Krzyżewska E, et al. Comparison of the phylogenetic analysis of PFGE profiles and the characteristic of virulence genes in clinical and reptile associated Salmonella strains. BMC Vet Res. 2019;15(1). DOI:10.1186/s12917-019-2019-1. PMC6721270 31477105

[47] Eetemadi A, Rai N, Pereira B, et al. The Computational Diet: a Review of Computational Methods Across Diet, Microbiome, and Health. Front Microbiol. 2020;11:393. eCollection 2020. 32318028 10.3389/fmicb.2020.00393 PMC7146706

[48] Boseret G, Losson B, Mainil JG, et al. Zoonoses in pet birds: review and perspectives. Vet Res. 2013;44(1):36. 23687940 10.1186/1297-9716-44-36 PMC3668993

[49] Franz R, Hummel J, Müller DW, et al. Herbivorous reptiles and body mass: effects on food intake, digesta retention, digestibility and gut capacity, and a comparison with mammals. Comp Biochem Physiol A Mol Integr Physiol. 2011;158(1):94–101. Epub 2010 Sep 17. 20850560 10.1016/j.cbpa.2010.09.007

[50] Sung YH, Hau BC, Karraker NE. Diet of the endangered big-headed turtle Platysternon megacephalum. PeerJ. 2016;4:e2784. 27994979 10.7717/peerj.2784 PMC5157187

[51] Jiang HY, Ma JE, Li J, et al. Diets alter the gut microbiome of crocodile lizards. Front Microbiol. 2017. DOI:10.3389/fmicb.2017.02073. PMC5660983 29118742

[52] Clancy MM, Davis M, Valitutto MT, et al. *Salmonella* infection and carriage in reptiles in a zoological collection. J Am Vet Med Assoc. 2016;248(9):1050–1059. 27074614 10.2460/javma.248.9.1050

[53] Grant RA, Montrose VT, Wills A. Wills AP.ExNOTic: should we be keeping exotic pets? Animals. 2017;7(12):47. 10.3390/ani7060047 PMC5483610 28629177

[54] Shomer NH, Holcombe H, Harkness JE. Biology and Diseases of Guinea Pigs. Laboratory Animal Medicine. 2015; 247–283. DOI:10.1016/B978-0-12-409527-4.00006-7.

[55] Ribas A, Saijuntha W, Agatsuma T, et al. Rodents as a Source of *Salmonella* Contamination in Wet Markets in Thailand. Vector Borne Zoonotic Dis. 2016;16(8):537–540. 27400325 10.1089/vbz.2015.1894 PMC4960473

[56] Lefebvre SL, Reid-Smith R, Boerlin P, et al. Evaluation of the risks of shedding Salmonellae and other potential pathogens by therapy dogs fed raw diets in Ontario and Alberta. Zoonoses Public Health. 2008;55(8–10):470–480. 18811908 10.1111/j.1863-2378.2008.01145.x

[57] Morley P, Strohmeyer R, Tankson J, et al. Evaluation of the association between feeding raw meat and *Salmonella enterica* infections at a Greyhound breeding facility. J Am Vet Med Assoc. 2006;228(10):1524–1532. 16677120 10.2460/javma.228.10.1524

[58] Finley R, Reid-Smith R, Ribble C, et al. The occurrence and antimicrobial susceptibility of Salmonellae isolated from commercially available canine raw food diets in three Canadian cities. Zoonoses Public Health. 2008. DOI:10.1111/j.1863-2378.2008.01147.x. 18811907

[59] Guyomard-Rabenirina S, Weill FX, Bastian S, et al. Reptiles in Guadeloupe (French West Indies) are a reservoir of major human *Salmonella enterica* serovars. PLoS One. 2019;14(7):e0220145. 10.1371/journal.pone.0220145 PMC6641201 31323053

[60] Frost D. Amphibian Species of the World: an Online Reference. American Museum of Natural History. 2016

[61] Ribas A, Poonlaphdecha S. Wild-Caught and Farm-Reared Amphibians are Important Reservoirs of Salmonella, A Study in North-East Thailand. Zoonoses Public Health. 2017;64(2):106-110. doi: 10.1111/zph.12286. 27359101

[62] Williams S, Patel M, Markey P, et al. *Salmonella* in the tropical household environment – everyday, everywhere. J Infect. 2015;71(6):642–648. 10.1016/j.jinf.2015.09.011 26416474

[63] Marenzoni ML, Zicavo A, Veronesi F, et al. Microbiological and parasitological investigation on chelonians reared in Italian facilities. Vet Ital. 2015;51(3):173–178. 26344661 10.12834/VetIt.7.21.3

[64] Sumiyama D, Shimizu A, Kanazawa T, et al. Prevalence of *Salmonella* in green anoles *(Anolis Carolinensis*), an invasive alien species in Naha and Tomigusuku Cities, Okinawa Main Island, Japan. J Vet Med Sci. 2020;82(5):678-680. doi:10.1292/jvms.19-0594. PMC7273597 32213730

[65] Lukac M, Pedersen K, Prukner-Radovcic E. Prevalence of *Salmonella* in captice reptiles from Croatia. J Zoo Wildl Med. 2015. DOI:10.1638/2014-0098r1.1. 26056873

[66] Bertelloni F, Chemaly M, Cerri D, et al. *Salmonella* infection in healthy pet reptiles: bacteriological isolation and study of some pathogenic characters. Acta Microbiol. Immunol Hung. 2016; DOI:10.1556/030.63.2016.2.5. 27352973

[67] Russo TP, Varriale L, Borrelli L, et al. *Salmonella* serotypes isolated in geckos kept in seven collections in southern Italy. J Small Anim Pract. 2018;59(5):294–297. 29315571 10.1111/jsap.12808

[68] Marin C, Lorenzo-Rebenaque L, Laso O, et al. Pet Reptiles: a Potential Source of Transmission of Multidrug-Resistant *Salmonella*. Front Vet Sci. 2021;7:613718.DOI: 10.3389/fvets.2020.613718. 10.3389/fvets.2020.613718 PMC7815585 33490138

[69] Nowakiewicz A, Ziółkowska G, Zięba P, et al. Aerobic Bacterial Microbiota Isolated from the Cloaca of The European Pond Turtle (*Emys orbicularis*) in Poland. J Wildl Dis. 2015;51(1):255–259. 25380369 10.7589/2013-07-157

[70] Pawlak A, Morka K, Bury S, et al. Microbiota in Free-Living Grass Snake Natrix natrix from Poland, Curr. Microbiol. 2020. DOI:10.1007/s00284-020-02021-3. PMC7415037 32424607

[71] Cota JB, Carvalho AC, Dias I, et al. *Salmonella* spp. in Pet Reptiles in Portugal: prevalence and Chlorhexidine Gluconate Antimicrobial Efficacy. Antibiotics. 2021;10(324):324. 10.3390/antibiotics10030324 PMC8003820 33808891

[72] Romero SB, Kvapil P, Cížek A, et al. The prevalence and antimicrobial resistance of Salmonella species isolated from captive reptiles at ljubljana zoo. Slov Vet Res. 2016;53 (1): 43-8.

[73] Jiménez RR, Barquero-Calvo E, Abarca JG, et al. Salmonella Isolates in the Introduced Asian House Gecko (Hemidactylus frenatus) with Emphasis on Salmonella Weltevreden, in Two Regions in Costa Rica. Vector-Borne Zoonotic Dis. 2015;15(9):550–555. 10.1089/vbz.2015.1785 26378974

[74] Zhang J, Kuang D, Wang F, et al. Turtles as a Possible Reservoir of Nontyphoidal Salmonella in Shanghai, China. Foodborne Pathog Dis. 2016;13(8):428–433. 10.1089/fpd.2015.2107 PMC5577352 27267492

[75] Abba Y, Ilyasu YM, Noordin MM. Isolation and identification of bacterial populations of zoonotic importance from captive non-venomous snakes in Malaysia. Microb Pathog. 2017;108:49–54. 10.1016/j.micpath.2017.04.038 28478198

[76] Abrahão CR, Moreno LZ, Silva JCR, et al. Salmonella enterica in Invasive Lizard from Fernando de Noronha Archipelago: serotyping, Antimicrobial Resistance and Molecular Epidemiology. Microorganisms. 2020 Dec 17;8(12):2017. 10.3390/microorganisms8122017 PMC7766374 33348534

[77] Ramos CP, Santana JA, Coura FM, et al.Identification and Characterization of *Escherichia coli, Salmonella* Spp., *Clostridium perfringens*, and *C. difficile* Isolates from Reptiles in Brazil. BioMed Res Int. (2019) 2019:9530732. DOI: 10.1155/2019/9530732. PMC6556801 31263711

[78] Whitten T, Bender JB, Smith K, et al. Reptile-associated salmonellosis in Minnesota, 1996-2011. Zoonoses Public Health. 2015, DOI: 10.1111/zph.12140. 24909385

[79] Martínez-Pérez P, Hyndman TH, Fleming PA. *Salmonella* in Free-Ranging Quokkas (*Setonix brachyurus)* from Rottnest Island and the Mainland of Western Australia. Animals (Basel). 2020;10(4):585. 10.3390/ani10040585 PMC7222713 32244325

[80] McWhorter A, Owens J, Valcanis M, et al. In vitro invasiveness and antimicrobial resistance of *Salmonella enterica* subspecies isolated from wild and captive reptiles. Zoonoses Public Health. 2021. DOI: 10.1111/zph.12820. 33655685

[81] Baling M, Mitchell C. Prevalence of *Salmonella* spp. in translocated wild reptiles and effect of duration of quarantine on their body condition. N Z Vet J. 2021;69(3):174–179. 33739909 10.1080/00480169.2021.1890647

[82] Rush EM, Amadi VA, Johnson R, et al. *Salmonella* serovars associated with Grenadian tree boa (Corallus grenadensis) and their antimicrobial susceptibility. Vet Med Sci. 2020;6(3):565–569. 31943909 10.1002/vms3.234 PMC7397926

[83] Prud’homme Y, Burton FJ, McClave C, et al. Prevalence, incidence and identification of Salmonella enterica from wild and captive grand cayman iguanas (*Cyclura lewisi*). J Zoo Wildl Med. 2018;49(4):959–966. 30592912 10.1638/2017-0234.1

[84] Kuroki R, Ishihara R, Nakajima N, et al. Prevalence of *Salmonella enterica* Subspecies enterica in Red-Eared Sliders Trachemys scripta elegans Retailed in Pet Shops in Japan. Jpn J Infect Dis. 2019;72(1):38–43. 30270249 10.7883/yoken.JJID.2018.140

[85] Krisjashahny *Notes from the Field*: Investigation of an Outbreak of *Salmonella* Paratyphi B Variant L(+) tartrate + (Java) Associated with Ball Python Exposure — USA, 2017. 10.15585/mmwr.mm6719a7 PMC6048943 29771878

[86] Gambino-Shirley K, Stevenson L, Concepción-Acevedo L, et al. Flea market finds and global exports: four multistate outbreaks of human *Salmonella* infections linked to small turtles, USA—2015, Zoonoses Public Health, 2018, DOI: 10.1111/zph.12466. 29577654

[87] Sundström K, Wahlström H, Ivarsson S, et al. Economic effects of introducing alternative *Salmonella* control strategies in Sweden. PLoS One. 2014;9(5):e96446. 10.1371/journal.pone.0096446 PMC4022667 24831797

[88] Ricard C, Mellentin J, Chabchoub RB, et al. Méningite à Salmonelle chez un nourrisson due à une tortue domestique. Arch Pediatr. 2015;22(6):605–607. 10.1016/j.arcped.2013.09.019 26014646

[89] Costa EP, Mam G, García LE. Los riesgos del empleo de reptiles como animales de compañía. Pediatr. Aten. Primaria. 2015; DOI:10.4321/s1139-76322015000300010.

[90] Horvath L, Kraft M, Fostiropoulos K, et al. *Salmonella enterica* subspecies diarizonae maxillary sinusitis in a snake handler: first report. Open Forum Infect Dis. 2016;3(2). DOI:10.1093/ofid/ofw066. PMC4866548 27186588

[91] Murphy D, Oshin F. Reptile-associated salmonellosis in children aged under 5 years in South West England. Arch Dis Child. 2015;100(4):364–365. 10.1136/archdischild-2014-306134 25538189

[92] Gavrilovici C, Pânzaru CV, Cozma S, et al. Message from a turtle: otitis with *salmonella arizonae* in children case report. Med (USA). 2017. DOI:10.1097/MD.0000000000008455. PMC5682812 29095293

[93] Baranzelli A, Loïez C, Bervar JF, et al. The snake raiser lung: an unusual cause of *Salmonella enterica* subspecies *arizonae* pneumoniaPoumons des éleveurs de serpents: une cause inhabituelle de pneumonie à *Salmonella enterica* subspecies *arizonae*. Med Mal Infect. 2017;47(6):424–425. 28602385 10.1016/j.medmal.2017.05.001

[94] Chen CY, Chen WC, Chin SC, et al. Prevalence and antimicrobial susceptibility of salmonellae isolates from reptiles in Taiwan. J Vet Diagnostic Investig. 2010;22(1):44–50. 10.1177/104063871002200107 20093681

[95] Jang YH, Lee SJ, Lim JG, et al. The rate of *Salmonella* spp. infection in zoo animals at Seoul Grand Park, Korea. J Vet Sci. 2008;9(2):177. 10.4142/jvs.2008.9.2.177 PMC2839095 18487939

[96] Kiebler CA, Bottichio L, Simmons L, et al. Outbreak of human infections with uncommon Salmonella serotypes linked to pet bearded dragons. Zoonoses Public Health. 2020;67(4):425–434. 32304287 10.1111/zph.12701 PMC11325769

[97] López-Quintana B, Rivas-González P, Toro-Rueda C, et al. Infección por *Salmonella enterica* subespecie salamae en un paciente ecuatoguineano consumidor de carne de tortuga. Enferm Infecc Microbiol Clin. 2015;33(6):430–431. 10.1016/j.eimc.2014.09.012 25466306

[98] Suzuki A, Tanaka T, Ohba K, et al. Purulent Pericarditis with *Salmonella enterica* Subspecies *arizona* in a Patient with Type 2 Diabetes Mellitus. Intern Med. 2017;56(16):2171–2174. 28781305 10.2169/internalmedicine.8293-16 PMC5596279

[99] Damborg P, Broens EM, Chomel BB, et al. Bacterial Zoonoses Transmitted by Household Pets: state-of-the-Art and Future Perspectives for Targeted Research and Policy Actions. J Comput Pathol. 2016;155(1):S27–S40. 10.1016/j.jcpa.2015.03.004 25958184

[100] Statista. Global dog and cat pet population 2018. https://www.statista.com/statistics/1044386/dog-and-cat-pet-population-worldwide/ (acessed 2021 Jul 05).

[101] Westgarth C, Pinchbeck GL, Bradshaw JW, et al. Factors associated with dog ownership and contact with dogs in a UK community. BMC Vet Res. 2017;3(1):5. 10.1186/1746-6148-3-5 PMC1852100 17407583

[102] Bataller E, García-Romero E, Llobat L, et al. Dogs as a source of *Salmonella* spp. in apparently healthy dogs in the Valencia Region. Could it be related with intestinal lactic acid bacteria? BMC Vet Res. 2020;16(1):268. 32746827 10.1186/s12917-020-02492-3 PMC7398315

[103] Cummings KJ, Mitchell PK, Rodriguez-Rivera LD, et al. Sequence analysis of *Salmonella enterica* isolates obtained from shelter dogs throughout Texas. Vet Med Sci. 2020;6(4):975–979. 32613739 10.1002/vms3.320 PMC7738724

[104] Reimschuessel R, Grabenstein M, Guag J, et al. Multilaboratory Survey To Evaluate *Salmonella* Prevalence in Diarrheic and Nondiarrheic Dogs and Cats in the USA between 2012 and 2014. J Clin Microbiol. 2017 May;55(5):1350–1368. 28202802 10.1128/JCM.02137-16 PMC5405253

[105] Amadi VA, Hariharan H, Arya G, et al. Serovars and antimicrobial resistance of non-typhoidal *Salmonella* isolated from non-diarrhoeic dogs in Grenada, West Indies. Vet Med Sci. 2017;4(1):26–34. 29468078 10.1002/vms3.84 PMC5813114

[106] Wu X, Angkititrakul S, Richards AL, et al. Risk of Antimicrobial Resistant Non-Typhoidal Salmonella during Asymptomatic Infection Passage between Pet Dogs and Their Human Caregivers in Khon Kaen, Thailand. Antibiotics (Basel). 2020;9(8):477. 10.3390/antibiotics9080477 PMC7460017 32759641

[107] Kim MW, Sharp CR, Boyd CJ, et al. Faecal PCR panel results and clinical findings in Western Australian dogs with diarrhoea. Aust Vet J. 2020;98(11):11. 32839975 10.1111/avj.13008

[108] Aeh P, Stayt J. The intestinal microbiome in dogs and cats with diarrhoea as detected by a faecal polymerase chain reaction-based panel in Perth, Western Australia. Aust Vet J. 2019;97(10):418–421. 31556108 10.1111/avj.12867 PMC7159723

[109] Kiflu B, Alemayehu H, Abdurahaman M, et al. *Salmonella* serotypes and their antimicrobial susceptibility in apparently healthy dogs in Addis Ababa, Ethiopia. BMC Vet Res. 2017;13(1):134. 28526020 10.1186/s12917-017-1055-y PMC5437602

[110] Vasco K, Graham JH, Trueba G. Detection of zoonotic enteropathogens in children and domestic animals in a semirural community in ecuador. Appl Environ Microbiol. 2016;82(14):4218–4224. 10.1128/AEM.00795-16 PMC4959199 27208122

[111] Castro KMN, Munoz ET, García GF, et al. Prevalence, risk factors, and identification of Salmonella spp. in stray dogs of northwest Mexico. Austral Journal of Veterinary Sciences. 2019;51:1.

[112] Askari N, Mashhad Rafiee S, Amini K. A case control study of *Salmonella* SPP. infection in stray dogs in Tehran shelters and the correlation between paraclinical tests results and clinical findings. Arch Razi Inst. 2020;75(1):93–99. 32292007 10.22092/ARI.2018.123213.1242 PMC8410164

[113] Botha WJ, Schoeman JP, Marks SL, et al. Prevalence of *Salmonella* in juvenile dogs affected with parvoviral enteritis. J S Afr Vet Assoc. 2018;89:1731. 10.4102/jsava.v89i0.1731 PMC6295970 30551702

[114] Whitfield K, Johnson L, Hobbs D, et al. Descriptive study of enteric zoonoses in Ontario, Canada, from 2010 – 2012. BMC Public Health, 2017, DOI: 10.1186/s12889-017-4135-9. PMC5320741 28222719

[115] Overgaauw PAM, Vinke CM, Van Hagen MAE, Paul AM, Overgaauw PAM, Vinke C, van Hagen MAE, Lipman LJA. A One Health. Perspective on the Human–Companion Animal Relationship with Emphasis on Zoonotic Aspects. Int J Environ Res Public Health. 2020;17(11):3789. 10.3390/ijerph17113789 PMC7312520 32471058

[116] Vercelli A, Lo Cicero E, Pazzini L. *Salmonella typhimurium* Endocarditis and Myocarditis in a Cat. Case Rep Vet Med. 2019;2019:7390530. 31886018 10.1155/2019/7390530 PMC6925778

[117] Vigo GB, Origlia J, Gornatti D, et al. Isolation of *Salmonella* Typhimurium from Dead Blue and Gold Macaws (*Ara ararauna)*. Avian Dis. 2009;53(1):135–138. 10.1637/8372-060208-Case.1 19432017

[118] Simpson KMJ, Hill-Cawthorne GA, Ward MP, et al. Diversity of *Salmonella* serotypes from humans, food, domestic animals and wildlife in New South Wales, Australia. BMC Infect Dis. 2018;18(1). DOI:10.1186/s12879-018-3563-1. PMC6280480 30518339

[119] Mather AI, Lawson B, De Pinna E, et al. Genomic Analysis of *Salmonella enterica* Serovar Typhimurium from Wild Passerines in England and Wales. Appl Environ Microbiol. 2016;82(22):6728–6735. DOI:10.1128/AEM.01660-16. 27613688 PMC5086566

[120] de Oliveira M, Camargo BQ, Cunha MSP, et al. Free-Ranging Synanthropic Birds (*Ardea alba* and *Columba livia domestica*) as Carriers of *Salmonella* spp. and Diarrheagenic *Escherichia coli* in the Vicinity of an Urban Zoo. Vector Borne Zoonotic Dis. 2018;18(1):65–69. DOI:10.1089/vbz.2017.2174. 29261025

[121] Torres-Mejía AM, Blanco-Peña K, Rodríguez C, et al. Zoonotic Agents in Feral Pigeons (*Columba livia*) from Costa Rica: possible Improvements to Diminish Contagion Risks. Vector Borne Zoonotic Dis. 2018;18(1):49–54. 29243991 10.1089/vbz.2017.2131

[122] Chomel BB. Diseases Transmitted by Less Common House Pets. Microbiol Spectr. 2015;3(6):6. 10.1128/microbiolspec.IOL5-0012-2015 27337276

[123] Himsworth CH, Zabek E, Desruisseau A, et al. PREVALENCE AND CHARACTERISTICS OF ESCHERICHIA COLI AND SALMONELLA SPP. IN THE FECES OF WILD URBAN NORWAY AND BLACK RATS (RATTUS NORVEGICUS AND RATTUS RATTUS) FROM AN INNER-CITY NEIGHBORHOOD OF VANCOUVER, CANADA. Journal of Wildlife Diseases. 2015;51(3):589–600. 10.7589/2014-09-242 25932669

[124] Sobrinho CP, JL G, Souza FN, et al. Prevalence of Diarrheagenic *Escherichia coli* (DEC) and *Salmonella* spp. with zoonotic potential in urban rats in Salvador, Brazil. Epidemiol Infect. 2020;149:e128, DOI:10.1017/S095026882000285X PMC8167902 33213546

[125] Pet Food Manufacter’s Association Annual Report. https://pfma-reports.co.uk/. (accessed 06 May 2021).

[126] Robertson S, Burakoff A, Stevenson L, et al. Recurrence of a Multistate Outbreak of *Salmonella* Enteritidis Infections Linked to Contact with Guinea Pigs — eight States, 2015–2017. MMWR Morb Mortal Wkly Rep. 2018;67(42):1195–1196. 30359346 10.15585/mmwr.mm6742a6 PMC6290818

[127] Collins J, Simpson KMJ, Bell G, et al. A One Health investigation of *Salmonella enterica* serovar Wangata in north-eastern New South Wales, Australia, 2016–2017. Epidemiol Infect. 2019;147:e150. 30869062 10.1017/S0950268819000475 PMC6518825

[128] Fearnley EJ, Lal A, Bates J, et al. *Salmonella* source attribution in a subtropical state of Australia: capturing environmental reservoirs of infection. Epidemiol Infect. 2018;146(15):1903–1908. 30103838 10.1017/S0950268818002224 PMC6452992

[129] Cooke FJ, De Pinna E, Maguire C, et al. First Report of Human Infection with Salmonella enterica Serovar Apapa Resulting from Exposure to a Pet Lizard. J Clin Microbiol. 2009;47(8):2672–2674. 10.1128/JCM.02475-08 PMC2725640 19535527

[130] Cummings KJ, Warnick LD, Elton M, et al. *Salmonella enterica* Serotype Cerro Among Dairy Cattle in New York: an Emerging Pathogen? Foodborne Pathog Dis. 2010;7(6):659–665. 10.1089/fpd.2009.0462 PMC3132111 20187753

[131] Altherr S, Lameter K. The Rush for the Rare: reptiles and Amphibians in the European Pet Trade. Animals (Basel). 2020;10(11):2085. 10.3390/ani10112085 PMC7697995 33182744

[132] Centers for Disease Control and Prevention, Healthy Pets, Healthy People https://www.cdc.gov/healthypets/specific-groups/veterinarians.html (accessed on 06 May 2021)

[133] van Roon M, Maas M, Toale D, et al. Live exotic animals legally and illegally imported via the main Dutch airport and considerations for public health. Plos One.2019;14(7):e0220122. DOI: 10.1371/journal.pone.0220122. PMC6655733 31339955

[134] Green J, Coulthard E, Norrey J, et al. Live Non-CITES Wildlife UK Imports and the Potential for Infectious Diseases. Animals (Basel). 2020;10(9):1632. 10.3390/ani10091632 PMC7552149 32932890

[135] Green J, Couthard E, Mehson D, et al. Blind trading: a literature review of research addressing the welfare of ball pythons in the exotic pet trade. Animals. 2020;10(2):193. 10.3390/ani10020193 PMC7070511 31979065

[136] Pulford CV, Wenner N, Redway ML, et al. The diversity, evolution and ecology of *Salmonella* in venomous snakes, PLoS Negl. Trop Dis. 2019;13(6):e0007169. 10.1371/journal.pntd.0007169 PMC6548357 31163033

[137] Warwick C, Steedman C. Wildlife-pet markets in a one-health context. Int J One Health. 2021;7(1):42–64.

[138] Nadimpalli ML, Pickering AJ. A call for global monitoring of WASH in wet markets. Lancet Planet Health. 2020;4(10):e439–e440. 33038315 10.1016/S2542-5196(20)30204-7 PMC7541042

[139] Santos P, Widmer KW, Rivera WL. PCR-based detection and serovar identification of Salmonella in retail meat collected from wet markets in Metro Manila. Philippines. PloS one. Vol. 15. 9 e0239457. 30 Sep 2020. 10.1371/journal.pone.0239457 PMC7526908 32997676

[140] Nhung NT, Van NTB, Cuong NV, et al. Antimicrobial residues and resistance against critically important antimicrobials in non-typhoidal *Salmonella* from meat sold at wet markets and supermarkets in Vietnam. Int J Food Microbiol. 2018 Feb 2;266: 301–309. 29275223 10.1016/j.ijfoodmicro.2017.12.015 PMC5783717

[141] Sripaurya B, Ngasaman R, Benjakul S, et al. Virulence genes and antibiotic resistance of *Salmonella* recovered from a wet market in Thailand. J Food Saf. 2019;39(2):e12601.

[142] Nadimpalli M, Fabre L, Yith V, et al. CTX-M-55-type ESBL-producing *Salmonella enterica* are emerging among retail meats in Phnom Penh, Cambodia. J Antimicrob Chemother. 2019;74(2):342–348. 30376113 10.1093/jac/dky451

[143] Stull JW, Peregrine AS, Sargeant JM, et al. Pet husbandry and infection control practices related to zoonotic disease risks in Ontario, Canada. BMC Public Health. 2013;13(1):520. 23714625 10.1186/1471-2458-13-520 PMC3668296

[144] Illegal wildlife trade https://www.wwf.sg/get_involved/illegal_wildlife_trade/ (accessed 06 May 2021)

[145] European Food Entry Authority; https://www.efsa.europa.eu/en/topics/topic/salmonella (accessed on 06 May 2021).

